# Evaluation of direct and maternal responses in reproduction traits based on different selection strategies for postnatal piglet survival in a selection experiment

**DOI:** 10.1186/s12711-021-00612-7

**Published:** 2021-03-15

**Authors:** Tuan Q. Nguyen, Pieter W. Knap, Geoff Simm, Sandra A. Edwards, Rainer Roehe

**Affiliations:** 1 Department of Agriculture, Horticulture and Engineering Sciences, SRUC (Scotland’s Rural College), Roslin Institute Building, Easter Bush Campus, Edinburgh, EH25 9RG Scotland, UK; 2 Department of Animal Breeding, Faculty of Animal Science and Veterinary Medicine, Nong Lam University – Ho Chi Minh City, Linh Trung Ward, Thu Duc District, Ho Chi Minh City, 71308 Vietnam; 3 Genus-PIC, 24837 Schleswig, Germany; 4 Global Academy of Agriculture and Food Security, Royal (Dick) School of Veterinary Studies, University of Edinburgh, Easter Bush Campus, Edinburgh, EH25 9RG Scotland, UK; 5 School of Natural and Environmental Sciences, Agriculture Building, Newcastle University, Newcastle upon Tyne, NE1 7RU UK

## Abstract

**Background:**

Postnatal piglet survival is important both in economic and animal welfare terms. It is influenced by the piglet’s own direct genetic effects and by maternal genetic effects of the dam, associated with milk production and mothering abilities. These genetic effects might be correlated, affected by other non-genetic factors and unfavourably associated with other reproduction traits such as litter size, which makes the development of optimal breeding strategies a challenge. To identify the optimum selection strategy for piglet survival, a selection experiment was carried out to compare responses in survival and reproduction traits to selection on only direct, only maternal, or both genetic effects of postnatal survival. The data of the experiment were recorded from outdoor reared pigs, with first- and second-generation sires selected based on their estimated breeding values for maternal and direct effects of postnatal survival of indoor reared offspring, respectively, with the opportunity to identify potential genotype-by-environment interaction.

**Results:**

A Bayesian multivariate threshold-linear model that was fitted to data on 22,483 piglets resulted in significant (Pr(h^2^ > 0) = 1.00) estimates of maternal and direct heritabilities between 0.12 and 0.18 for survival traits and between 0.29 and 0.36 for birth weight, respectively. Selection for direct genetic effects resulted in direct and maternal responses in postnatal survival of 1.11% ± 0.17 and − 0.49% ± 0.10, respectively, while selection for maternal genetic effects led to greater direct and maternal responses, of 5.20% ± 0.34 and 1.29% ± 0.20, respectively, in part due to unintentional within-litter selection. Selection for both direct and maternal effects revealed a significant lower direct response (− 1.04% ± 0.12) in comparison to its expected response from single-effect selection, caused by interactions between direct and maternal effects.

**Conclusions:**

Selection successfully improved post- and perinatal survival and birth weight, which indicates that they are genetically determined and that genotype-by-environment interactions between outdoor (experimental data) and indoor (selection data) housed pigs were not important for these traits. A substantially increased overall (direct plus maternal) response was obtained using selection for maternal *versus* direct or both direct and maternal effects, suggesting that the maternal genetic effects are the main limiting factor for improving piglet survival on which selection pressure should be emphasized.

## Background

Piglet mortality is one of the most commercially-important traits in pig production systems [1–3] and leads to animal welfare concerns [4] and economic losses [3, 5, 6]. Based on a recent report, the average rate of stillborn piglets for all breeding herds in the UK was 5.1% and the mean pre-weaning mortality was 12.1% [7]. Selection schemes that focus only on a reproduction trait such as litter size and on productivity traits such as growth rate and lean meat, result in reduced piglet survival due to undesirable genetic correlations between these traits [8–10]. Piglet birth weight is an important factor associated with piglet survival [11]. The low birth weight of piglets has been shown to be under the influence of intrauterine growth restriction, which results in physiological immaturity and dysfunction of organs and tissues that are important to digestion, nutrient absorption, and metabolism [12–14].

Piglet survival is controlled both by the genes of the piglet that are involved in vitality, health, growth, etc. (direct genetic effects), and by the genes of the dam that affect milk yield and other mothering abilities (maternal genetic effects), which is a challenge for improving piglet survival genetically [15]. In the literature, direct and maternal heritabilities for piglet survival are reported to be low, but the genetic variation is sufficient to obtain a meaningful selection response [16–18]. Genetic parameters of piglet survival have been estimated based on the performance of sows (assuming normally-distributed continuous traits) by fitting a linear model using restricted maximum likelihood (REML) [19, 20]. However, in order to estimate the direct and maternal heritabilities and the genetic correlations between direct and maternal effects of piglet survival traits and birth weight, the data have to be analysed at the individual piglet level. At the piglet level, observations of survival are binary (alive or dead), which are more appropriately analysed using a threshold model [21]. A Bayesian approach is particularly appropriate for joint analysis of binary and normally distributed traits, using a combined threshold-linear model [22].

Using data from a large selection experiment for postnatal survival, the aim of this study was to estimate direct and maternal selection responses of piglet survival and birth weight at the piglet level, using a Bayesian threshold-linear model, as well as the direct selection responses of survival, reproduction, and birth weight traits at the sow level, using a linear model. These same models were used to estimate the direct and maternal genetic parameters of piglet survival and birth weight at the piglet level and of piglet survival, reproduction, and birth weight traits at the sow level. Cross-classified mating between animals from direct and maternal selection groups allowed us to estimate responses when selection was for maternal or direct genetic effects only or for a combination of both. The selection experiment also allowed us to investigate whether genotype-by-environment interactions occurred because the selection of boars was based on direct and/or maternal estimated breeding values (EBV) for postnatal piglet survival from an indoor production system, while the sows and piglet performances were obtained in an outdoor system.

## Methods

### Animals and data

Data were available on 22,483 piglets born in 1765 litters from an outdoor selection experiment carried out over two generations. In the first-generation, 28 Landrace boars (from a dam line) were selected for high or average maternal EBV for piglet postnatal survival. The EBV for piglet survival of the boars were estimated, in 2004–2005, by the Pig Improvement Company (PIC) based on postnatal survival of indoor reared piglets using a linear direct-maternal effects model. The selected boars were randomly mated with 413 commercial dams to produce 567 gilts, with 280 gilts sired by high maternal EBV boars and 287 gilts sired by average maternal EBV boars. In the second-generation, these two groups of gilts were mated with two groups of Large White boars of a sire line (29 in total). The Large White boars were selected for high or average EBV for direct genetic effects for postnatal survival. The sows in the second generation were kept for three parities. In order to investigate and disentangle direct and maternal genetic effects, all combinations of high and average EBV of maternal and direct genetic effects were planned, which meant that a cross-classified mating scheme was applied to guarantee that every second-generation sow was mated at least once with Large White boars with high *versus* average direct EBV.

Two sets of data were used in this study. The first dataset comprised performance records from second and third generation outdoor reared individual piglets which included survival at birth (SVB), also referred to as perinatal survival in this paper, survival during the nursing period (SVNP), also referred to as postnatal survival, and individual birth weight (IBW). Survival of piglets was recorded on a binary scale, 1 for stillborn, 2 for alive. For SVB, records on 22,483 piglets were available, with 21,669 alive and 814 stillborn piglets. Mummified piglets were excluded from the analysis. Stillborn piglets were then treated as missing values for the trait SVNP (with 19,197 alive and 2472 dead piglets). Each individual piglet, regardless of whether it was stillborn or alive, was weighed within 24 h after birth. Cross-fostering was operated from first handling up to 4 days after farrowing. Only 8.2% of the total number of piglets was cross-fostered. The second dataset contained performance records at the sow level: number of piglets born (NB), average piglet birth weight per litter (ABW), the standard deviation of piglet birth weight within litter (SDBW), piglet survival rate per litter at birth (SVLB), and piglet survival rate per litter during the nursing period (SVLNP). All litter weight traits included the weights of both the stillborn and live piglets.

### Statistical analysis

For the binary traits SVB and SVNP, a threshold model was used, in which the traits were considered to have an underlying continuous distribution (liability). The threshold is a conceptual point estimated for the binary observations. If the animal’s liability value is above the threshold, it is considered as having “survived” and if it is below the threshold, it is considered as “dead” [23]. In contrast, for birth weight, a linear model was fitted by considering a normal distribution.

#### Analysis at the piglet level

At the piglet level, a Bayesian threshold-linear model was fitted in a multivariate analysis of the traits SVB, SVNP, and IBW, as follows:

$${\mathbf{y}} ={\mathbf{Xb}} + {\mathbf{Z}}_{1} {\mathbf{d}} + {\mathbf{Z}}_{2} {\mathbf{m}} + {\mathbf{Z}}_{3} {\mathbf{l}} + {\mathbf{e}},$$

where ${\mathbf{y}}$ is a vector that includes the underlying continuous variables for SVB and SVNP, and the phenotypic values for IBW; ${\mathbf{b}}$ is a vector of fixed effects, i.e. the effects of farm-unit-year-month-parity (23 classes), gestation length (9 classes), and sex of piglets (2 classes), along with a fostering effect (2 classes) (for SVNP only), for whether a piglet stayed with its biological mother or was transferred to a nursing sow; vectors ${\mathbf{d}}$, ${\mathbf{m}}$, and ${\mathbf{l}}$ represent the random effects of direct genetic, maternal genetic, and common environmental litter effects, respectively; ${\mathbf{X}}$, ${\mathbf{Z}}_{1}$, ${\mathbf{Z}}_{2}$, and ${\mathbf{Z}}_{3}$ are incidence matrices relating vectors ${\mathbf{b}}$, ${\mathbf{d}}$, ${\mathbf{m}}$, and ${\mathbf{l}}$, respectively, with the observations.The following (co)variance structure analyses were assumed in the fitted model:

$${\mathbf{V}}\left[ {\begin{array}{*{20}c} {\mathbf{d}} \\ {\mathbf{m}} \\ {\mathbf{l}} \\ {\mathbf{e}} \\ \end{array} } \right] = \left[ {\begin{array}{*{20}c} {{\mathbf{A}} \otimes {\mathbf{G}}_{{\mathbf{d}}} } & {{\mathbf{A}} \otimes {\mathbf{G}}_{{{\mathbf{dm}}}} } & 0 & 0 \\ {{\mathbf{A}} \otimes {\mathbf{G}}_{{{\mathbf{md}}}} } & {{\mathbf{A}} \otimes {\mathbf{G}}_{{\mathbf{m}}} } & 0 & 0 \\ 0 & 0 & {{\mathbf{I}} \otimes {\mathbf{L}}} & 0 \\ 0 & 0 & 0 & {{\mathbf{I}} \otimes {\mathbf{R}}} \\ \end{array} } \right],$$

where $\otimes$ is the direct product of matrices, ${\mathbf{A}}$ is the numerator genetic relationship matrix (four generation deep) at the piglet level, and ${\mathbf{G}}_{{\mathbf{d}}}$, ${\mathbf{G}}_{{\mathbf{m}}}$, ${\mathbf{G}}_{{{\mathbf{dm}}}}$, ${\mathbf{L}}$, and ${\mathbf{R}}$ represent the (co)variance matrices between traits for direct genetic, maternal genetic, associations between direct and maternal genetic effects, litter, and residual effects, respectively. For estimation of the maternal effects of postnatal survival, the biological mother was also considered for cross-fostered piglets because of the relatively long cross-fostering period of up to four days after birth and the low percentage of cross-fostering.

To estimate the responses to selection at the piglet level, the analysis of genetic parameters that was previously carried out by Roehe et al. [24] on the same data was repeated because of the availability of an updated version of the software, of a substantially larger number of Gibbs sampling iterations with a longer burn-in period, and of additional pedigree information. In a further analysis, piglet survival traits and birth weight were adjusted for litter size by adding litter size as a covariate, in order to investigate its influence on the parameters and on selection responses in direct and maternal genetic effects of these adjusted traits.

#### Analysis at the sow level

At the sow level, the data were analysed using a multiple trait Bayesian linear model for NB, ABW, SDBW, SVLB, and SVLNP. NB included total counts of live and stillborn piglets, but not mummified piglets. To increase the accuracy of SDBW, 1735 litters with five or more piglets were used in the analysis. SVLB was calculated as the number of piglets born alive divided by the total number of piglets born. SVLNP was obtained by dividing the number of piglets surviving the nursing period by the number of piglets born alive.

The multiple trait model used to estimate genetic parameters for sow reproduction performance was:

$${\mathbf{y}} = {\mathbf{Xb}} + {\mathbf{Z}}_{1} {\mathbf{a}} + {\mathbf{Z}}_{2} {\mathbf{pe}} + {\mathbf{e}},$$

where ${\mathbf{y}}$, ${\mathbf{b}}$, ${\mathbf{a}}$, ${\mathbf{pe}}$ and ${\mathbf{e}}$ are vectors of the observations of traits, fixed effects (farm-unit-year-month-parity (23 classes), gestation length (9 classes), and generation (2 classes)), random additive genetic effects of sows, random permanent environmental effects, and residuals, respectively, and ${\mathbf{X}}$, ${\mathbf{Z}}_{1}$ and ${\mathbf{Z}}_{2}$ are incidence matrices relating vectors ${\mathbf{b}}$, ${\mathbf{a}}$,and ${\mathbf{pe}}$ with ${\mathbf{y}}$. The assumed (co)variance structure was:

$${\mathbf{V}}\left[ {\begin{array}{*{20}c} {\mathbf{a}} \\ {\mathbf{l}} \\ {\mathbf{e}} \\ \end{array} } \right] = \left[ {\begin{array}{*{20}c} {{\mathbf{A}} \otimes {\mathbf{G}}} & 0 & 0 \\ 0 & {{\mathbf{I}} \otimes {\mathbf{PE}}} & 0 \\ 0 & 0 & {{\mathbf{I}} \otimes {\mathbf{R}}} \\ \end{array} } \right],$$

where ${\mathbf{A}}$ is the numerator genetic relationship matrix at the sow level and ${\mathbf{G}}$, ${\mathbf{PE}},$ and ${\mathbf{R}}$ represent the (co)variance matrices between traits for direct genetic effects of sows, permanent environmental effects, and residual effects, respectively.

#### Estimation of variance components

All Bayesian analyses were performed using the program THRGIBBS1f90 [22]. Convergence of the parameters was assessed by Geweke’s diagnostic value [25] and by visualisation of the mixing of the Markov chain using a trace plot. At the piglet level, the Gibbs sampling process for estimation of variance components was run for 1,000,000 iterations and the first 500,000 were deleted as burn-in based on visual evaluation. At the sow level, the Gibbs sampling process was allowed to run for 2,000,000 iterations and the first 1,200,000 iterations were discarded as burn-in. To avoid autocorrelation, only the values from every 30th iteration and every 50th iteration for piglet and sow analyses, respectively, were stored and used to calculate the marginal posterior distribution of the parameters. Estimates of variance components, heritabilities, and correlations were calculated as the means of their corresponding marginal posterior distributions and their credibility was provided as the 95% highest posterior density interval (95-HPD).

The variance components for direct genetic effects (${\varvec{\upsigma}}_{{\mathbf{d}}}^{2}$), maternal genetic effects (${\varvec{\upsigma}}_{{\mathbf{m}}}^{2}$), the covariance between direct and maternal genetic effects (${\varvec{\upsigma}}_{{{\mathbf{dm}}}}$), litter effects (${\varvec{\upsigma}}_{{\mathbf{l}}}^{2}$), and residuals (${\varvec{\upsigma}}_{{\mathbf{e}}}^{2}$) were used to calculate the phenotypic variance at the piglet level [26] as:

$${\varvec{\upsigma}}_{{\mathbf{P}}}^{2} = {\varvec{\upsigma}}_{{\mathbf{d}}}^{2} + {\varvec{\upsigma}}_{{\mathbf{m}}}^{2} + {\varvec{\upsigma}}_{{{\mathbf{dm}}}} + {\varvec{\upsigma}}_{{\mathbf{l}}}^{2} + {\varvec{\upsigma}}_{{\mathbf{e}}}^{2} .$$

This variance was used as basis for estimating the direct and maternal heritabilities of piglet traits.

The variance components for additive genetic effects (${\varvec{\upsigma}}_{{\mathbf{a}}}^{2}$), permanent environmental effects (${\varvec{\upsigma}}_{{{\mathbf{pe}}}}^{2}$), and residuals (${\varvec{\upsigma}}_{{\mathbf{e}}}^{2}$) were used to calculate the phenotypic variance at the sow level as:

$${\varvec{\upsigma}}_{{{\mathbf{Ps}}}}^{2} = {\varvec{\upsigma}}_{{\mathbf{a}}}^{2} + {\varvec{\upsigma}}_{{{\mathbf{pe}}}}^{2} + {\varvec{\upsigma}}_{{\mathbf{e}}}^{2} .$$

This variance was used as a basis to estimate the heritabilities of sow productivity traits.

#### Estimation of responses to selection

The responses to selection were estimated based on EBV generated based on the models described before using Gibbs sampling, keeping the variance components matrices fixed at the values obtained in the genetic parameter analyses. The Gibbs sampling algorithm was run for 20,000 iterations, with the first 10,000 iterations deleted as burn-in and the solutions of every 30^th^ iteration saved for both the piglet and sow analyses. For the survival traits, the EBV were on the liability scale and were transformed to the phenotypic probability scale using:

$${\text{p}}_{\text{i}} = \Phi \left( {\upmu + {\text{EBV}}_{\text{i}} } \right),$$

where ${\text{p}}_{\text{i}}$ is the probability for survival of piglet ${\text{i}}$, $\Phi \left( . \right)$ is the cumulative probability function of the standard normal distribution, $\upmu$ is the liability of the mean of the respective trait, and ${\text{EBV}}_{\text{i}}$ is the EBV of piglet ${\text{i}}$ on the liability scale [27].

At the piglet level, the selection response was estimated as the difference between EBV of the high (${\text{H}}$) and control (${\text{C}}$) groups of third-generation piglets, which were grouped based on their maternal Landrace grandsires belonging to the high (${\text{H}}_{\text{M}}$) or control $({\text{C}}_{\text{M}}$) group for maternal (${\text{M}}$) genetic effects of postnatal survival and based on their Large White sires belonging to the high (${\text{H}_\text{D} )}$ or control (${\text{C}_\text{D}}$) group for direct (${\text{D}}$) genetic effects of postnatal survival. Due to the cross-classified mating design, the third-generation piglets were assigned into four selection groups: ${{\text{C}}_{\text{D}}} {{\text{C}}_{\text{M}}}$, ${{\text{H}_\text{D}}} {{\text{C}_\text{M}} }$, ${\text{C}_\text{D}} {\text{H}_\text{M} }$, and ${{\text{H}}_{\text{D}}} {{\text{H}}_{\text{M}} }$, representing the control group originated from average EBV sires, and the selection groups for direct effects, maternal genetic effects, and selection on both genetic effects using a tandem selection strategy, respectively.

Based on the design of the selection experiment, the genetic contribution of each ancestor group to the selection response (R) based on the direct EBV of the third-generation piglets estimated based on the experimental data can be modelled as:

$$\text{R}\left( {{\text{EBV}}_{\text{d}} } \right) = 1/2{\text{LW}}_{\text{d}} + 1/4{\text{LR}}_{\text{d}} + 1/4{\text{CS}}_{\text{d}} + 1/2{\text{LW}}_{\text{dm}} + 1/4{\text{LR}}_{\text{dm}} + 1/4{\text{CS}}_{\text{dm}} ,$$

where ${\text{d}}$ and ${\text{dm}}$ represent the direct and correlated direct-maternal effects for the Large White (LW) boars, the Landrace (LR) boars, and the commercial sows (CS) [28]. Similarly, the contribution of each ancestor group to selection response based on the maternal EBV of the third-generation piglets estimated based on the experimental data can be modelled as:

$${\text{R}}\left( {{\text{EBV}}_{\text{m}} } \right) = 1/2{\text{LW}}_{\text{m}} + 1/4{\text{LR}}_{\text{m}} + 1/4{\text{CS}}_{\text{m}} + 1/2{\text{LW}}_{\text{md}} + 1/4{\text{LR}}_{\text{md}} + 1/4{\text{CS}}_{\text{md}} .$$

Thus, the selection response for direct genetic effects $\left( {\Delta_{\text{D}} } \right)$ based on the means of direct EBV $\left( {{\text{EBV}}_{\text{d}} } \right)$ of third-generation piglets for the different selection groups is:${{{\text{p}}}{\Delta_{{{\text{D}}}}\left({{\text{H}}_{\text{D}} {{\text{C}}_{\text{M}}} - {{\text{C}}_{{\text{D}}}} {{\text{C}}_{{\text{M}}} }} \right)}} = {{\text{EBV}}_{{{\text{d}}\left( {{\text{H}}_{{\text{D}}} {{\text{C}}_{{\text{M}}}} } \right)}} - {{\text{EBV}}}_{{{\text{d}}\left( {{\text{C}}_{{\text{D}}} {{\text{C}}_{{\text{M}}} }} \right)}} }= {{\text{pLW}}_{\text{d}}}$ with genetic contribution ${\text{p}} = 1/2$,

1 $$\Delta_{{{\text{D}}\left( {{\text{H}}_{\text{D}} {{\text{C}}_{\text{M}}} - {{\text{C}}_{{\text{D}}}} {{\text{C}}_{{\text{M}}} }} \right)}} = 2\left( {{\text{EBV}}_{{{\text{d}}\left( {{\text{H}}_{{\text{D}}} {{\text{C}}_{{\text{M}}}} } \right)}} - {{\text{EBV}}}_{{{\text{d}}\left( {{\text{C}}_{{\text{D}}} {{\text{C}}_{{\text{M}}} }} \right)}} } \right).$$

${\text{p}}\Delta_{{{\text{D}}\left( {{\text{C}}_{{\text{D}}} {{\text{H}}_{{\text{M}}}} - {{\text{C}}_{{\text{D}}}} {{\text{C}}_{\text{M}}}} \right)}} = {{\text{EBV}}_{{{\text{d}}\left( {{\text{C}}_{{\text{D}}} {{\text{H}}_{{\text{M}}}} } \right)}} - {{\text{EBV}}}_{{{\text{d}}\left( {{\text{C}}_{{\text{D}}} {{\text{C}}_{{\text{M}}} }} \right)}} } = {{\text{pLR}}_{\text{dm}}}$ with genetic contribution ${\text{p}} = 1/4$,

2 $$\Delta_{{{\text{D}}\left( {{\text{C}}_{{\text{D}}} {{\text{H}}_{\text{M}}} - {{\text{C}}_{{\text{D}}}} {{\text{C}}_{{\text{M}}}} } \right)}} = 4\left( {{\text{EBV}}_{{{\text{d}}\left( {{\text{C}}_{{\text{D}}} {{\text{H}}_{{\text{M}}}} } \right)}} - {{\text{EBV}}}_{{{\text{d}}\left({{{\text{C}}_{{\text{D}}}} {{\text{C}}_{\text{M}}} } \right)}} } \right).$$

$$\Delta_{{{\text{D}}\left( {{\text{H}}_{\text{D}} {{\text{H}}}_{{\text{M}}} - {{\text{C}}}_{{\text{D}}} {{\text{C}}}_{{\text{M}}} } \right)}} = {\text{EBV}}_{{{\text{d}}\left( {{\text{H}}_{{\text{D}}} {{\text{H}}}_{{\text{M }}} } \right)}} - {{\text{EBV}}}_{{{\text{d}}({{\text{C}}}_{{\text{D}}} {{\text{C}}}_{{{\text{M}})}} }} = {{{1/2 \text{LW}}}}_{{\text{d}}} + {{{1/4 {\text{LR}}}}}_{{\text{dm}}} + \Delta_{{{\text{Dd}} \times {{\text{m}}}}} ,$$

where $\Delta_{{{\text{Dd}} \times {{\text{m}}}}}$ is the deviation of the direct response from the expected response obtained for single-effect (direct or maternal) selection when selection was for both direct and maternal effects, estimating the influence of interactions between these effects on the direct response to selection:

3 $$\Delta_{{{\text{Dd}} \times {{\text{m}}}}} = \left( {{\text{EBV}}_{{{\text{d}}\left( {{\text{H}}_{{\text{D}}} {{\text{H}}}_{{\text{M}}} } \right)}} - {{\text{EBV}}}_{{{\text{d}}\left( {{\text{C}}_{{\text{D}}} {\text{C}}_{{\text{M}}} } \right)}} } \right) - \left( {{{1/2 {\text{LW}}}}_{{\text{d}}} + 1/4{{LR}}_{{\text{dm}}} } \right).$$

The selection response for maternal genetic effect $\left( {\Delta_{\text{M}} } \right)$ based on maternal EBV $\left( {{\text{EBV}}_{\text{m}} } \right)$ of third-generation piglets for the different selection groups is:

${{\text{p}}}\Delta_{{{\text{M}}\left( {{\text{H}}_{{\text{D}}} {{\text{C}}}_{{\text{M}}} - {{\text{C}}}_{{\text{D}}} {{\text{C}}}_{{\text{M}}} } \right)}} = {{\text{EBV}}}_{{{\text{m}}\left( {{\text{H}}_{{\text{D}}} {{\text{C}}}_{{\text{M}}} } \right)}} - {{\text{EBV}}}_{{{\text{m}}\left( {{\text{C}}_{{\text{D}}} {{\text{C}}}_{{\text{M}}} } \right)}} = {{\text{pLW}}}_{{\text{md}}}$ with genetic contribution ${\text{p}} =1/2$,

4 $$\Delta_{{{\text{M}}\left( {{\text{H}}_{{\text{D}}} {{\text{C}}}_{{\text{M}}} - {{\text{C}}}_{{\text{D}}} {{\text{C}}}_{{\text{M}}} } \right)}} = 2({{\text{EBV}}}_{{{\text{m}}\left( {{\text{H}}_{{\text{D}}} {{\text{C}}}_{{\text{M}}} } \right)}} - {{\text{EBV}}}_{{{\text{m}}\left( {{\text{C}}_{{\text{D}}} {{\text{C}}}_{{\text{M}}} } \right)}} ).$$

$${\text{p}}\Delta_{{{\text{M}}\left( {{\text{C}}_{\text{D}} {{\text{H}}}_{{\text{M}}} - {{\text{C}}}_{\text{D}} {{\text{C}}}_{{\text{M}}} } \right)}} = {\text{EBV}}_{{{\text{m}}\left( {{\text{C}}_{{\text{D}}} {{\text{H}}}_{{\text{M}}} } \right)}} - {{\text{EBV}}}_{{{\text{m}}\left( {{\text{C}}_{{\text{D}}} {{\text{C}}}_{{\text{M}}} } \right)}} = {\text{pLR}}_{\text{m}}$$ with genetic contribution $${{{\text{p} = \raise.5ex\hbox{$\scriptstyle 1$}\kern-.1em/ \kern-.15em\lower.25ex\hbox{$\scriptstyle 4$} }}}$$,

5 $$\Delta_{{{\text{M}}\left( {{\text{C}}_{{\text{D}}} {{\text{H}}}_{{\text{M}}} - {{\text{C}}}_{{\text{D}}} {{\text{C}}}_{{\text{M}}} } \right)}} = 4({\text{EBV}}_{{{\text{m}}\left( {{\text{C}}_{{\text{D}}} {{\text{H}}}_{{\text{M}}} } \right)}} - {{\text{EBV}}}_{{{\text{m}}\left( {{\text{C}}_{{\text{D}}} {{\text{C}}}_{{\text{M}}} } \right)}} ) .$$

$$\Delta_{{{\text{M}}\left( {{\text{H}}_{{\text{D}}} {{\text{H}}}_{{\text{M}}} - {{\text{C}}}_{{\text{D}}} {{\text{C}}}_{{\text{M}}} } \right)}} = {{\text{EBV}}}_{{{\text{m}}\left( {{\text{H}}_{{\text{D}}} {{\text{H}}}_{{\text{M}}} } \right)}} - {{\text{EBV}}}_{{{\text{m}}\left( {{\text{C}}_{{\text{D}}} {{\text{C}}}_{{\text{M}}} } \right)}} = 1/2{{\text{ LW}}}_{{\text{md}}} + 1/4{\text{ LR}}_{{\text{m}}} + \Delta_{{{\text{Md}} \times {\text{m}}}} ,$$

where $\Delta_{{{\text{Md}} \times {\text{m}}}}$ is the deviation of the maternal response from the expected response obtained for single-effect (direct or maternal) selection when selection was for both direct and maternal effects, estimating the influence of interactions between these effects on the maternal response to selection:

6 $$\Delta_{{{\text{Md}} \times {{\text{m}}}}} = ({{\text{EBV}}}_{{{\text{m}}\left( {{\text{H}}_{{\text{D}}} {{\text{H}}}_{{\text{M}}} } \right)}} - {{\text{EBV}}}_{{{\text{m}}\left( {{\text{C}}_{{\text{D}}} {{\text{C}}}_{{\text{M}}} } \right)}} ) - \left( {{\text{{1/2LW}}}_{{\text{md}}} + {{\text{1/4LR}}}_{{\text{m}}} } \right) .$$

Equations (1) to (6) were used to estimate the direct and maternal responses for the different selection scenarios using the EBV of the third-generation piglets.

At the sow level, selection responses $\left( {{\text{R}}_{\text{S}} } \right)$ were estimated as differences between mean maternal EBV of the maternal selection (${\text{H}}_{\text{M}}$) and control (${\text{C}}_{\text{M}}$) groups of the second-generation crossbred sows:

$${\text{R}}_{\text{S}} = 2\left( {{\text{EBV}}_{{{\text{m}}\left( {{\text{H}}_{\text{M}} } \right)}} - {\text{EBV}}_{{{\text{m}}\left( {{\text{C}}_{\text{M}} } \right)}} } \right).$$

## Results

Table 1 summarises the performance parameters of piglets and sows from the two-generation selection experiment. In total, 980 sows produced 1765 litters, comprising 22,483 piglets. The average piglet birth weight was 1.600 kg, with a standard deviation (SD) of 401 g. The corresponding coefficient of variation of 25% was equal to that for total number of piglets born and number of piglets born alive.

**Table 1:** Number of observations, means, standard deviations (SD) and coefficients of variation (CV) for piglet and sow performance traits

Trait	Number of observations	Mean	SD	CV
Individual piglet birth weight (kg)	22,107	1.600	0.401	0.25
Piglet survival rate per litter at birth (%)	1765	96.65	6.97	0.07
Piglet survival rate per litter during the nursing period (%)	1765	89.03	11.96	0.13
Average piglet birth weight per litter (kg)	1765	1.636	0.272	0.17
SD of piglet birth weight within litter (kg)	1765	0.312	0.096	0.31
Total number piglets born	1765	12.74	3.19	0.25
Number piglets born alive	1765	12.28	3.08	0.25

### Variance components at the piglet level

The variance components of the data at the piglet level were reanalysed with updated software and a substantially larger number of iterations including a longer burn-in period, which resulted in similar heritability estimates and similar low estimates of genetic correlations between direct and maternal effects of the piglet survival traits and birth weight (Table 2) to those reported in Roehe et al. [24]. The main difference between these two analyses was that the antagonistic genetic correlations between direct and maternal effects within trait were moderate in the previous analysis, but only slightly antagonistic in the current study with estimates of − 0.15, − 0.04, and − 0.05 for SVNP, SVB, and IBW, respectively, and a probability of being negative of 95, 63, and 83%, respectively. Due to the use of a substantially larger number of Gibbs sampling iterations in the current study, including a longer burn-in, we found that more of the low correlations between traits were significant. For example, estimates of the direct or maternal genetic correlations between piglet traits were all significant but low, i.e. ranging from 0.23 to 0.25 for direct effects and from 0.18 to 0.28 for maternal effects. In addition, estimates of some correlations between direct and maternal genetic effects of different traits were significant, in particular the correlations between ${\text{IBW}}_{\text{d}}$ and ${\text{SVB}}_{\text{m}}$ (0.16), between ${\text{IBW}}_{\text{d}}$ and ${\text{SVNP}}_{\text{m}}$ (0.15) and between ${\text{SVNP}}_{\text{d}}$ and ${\text{IBW}}_{\text{m}}$ (0.14). Adjustment of the piglet traits for litter size resulted in negligible changes in estimates of genetic parameters compared to the unadjusted analysis (see Additional file 1: Table S1).

**Table 2:** Estimated genetic parameters for piglet survival traits and birth weight analysed at the piglet level

Effect	Trait	Genetic variance	Direct			Maternal		
			SVB	SVNP	IBW	SVB	SVNP	IBW
Direct	SVB	0.335	0.178*	0.225*	0.230*	− 0.041	0.146	0.119
			(0.13 to 0.23)	(− 0.00 to 0.44)	(0.11 to 0.35)	(− 0.26 to 0.17)	(− 0.05 to 0.34)	(− 0.00 to 0.24)
			Pr(h^2^ > 0) = 1.00	Pr(r_g_ > 0) = 0.97	Pr(r_g_ > 0) = 1.00	Pr(r_g_ < 0) = 0.63	Pr(r_g_ > 0) = 0.93	Pr(r_g_ > 0) = 0.97
	SVNP	0.287	0.069	0.184*	0.254*	0.165	– 0.152	0.137*
				(0.13 to 0.24)	(0.14 to 0.37)	(− 0.04 to 0.37)	(− 0.33 to 0.03)	(0.01 to 0.26)
				Pr(h^2^ > 0) = 1.00	Pr(r_g_ > 0) = 1.00	Pr(r_g_ > 0) = 0.94	Pr(r_g_ < 0) = 0.95	Pr(r_g_ > 0) = 0.98
	IBW	0.091	0.04	0.04	0.358*	0.163*	0.149*	– 0.047
					(0.32 to 0.40)	(0.04 to 0.29)	(0.03 to 0.27)	(− 0.14 to 0.05)
					Pr(h^2^ > 0) = 1.00	Pr(r_g_ > 0) = 0.99	Pr(r_g_ > 0) = 0.99	Pr(r_g_ < 0) = 0.83
Maternal	SVB	0.261	− 0.015	0.045	0.025	0.140*	0.233*	0.175*
						(0.11 to 0.17)	(0.07 to 0.40)	(0.06 to 0.29)
						Pr(h^2^ > 0) = 1.00	Pr(r_g_ > 0) = 1.00	Pr(r_g_ > 0) = 1.00
	SVNP	0.181	0.035	− 0.037	0.019	0.0507	0.117*	0.278*
							(0.09 to 0.14)	(0.18 to 0.38)
							Pr(h^2^ > 0) = 1.00	Pr(r_g_ > 0) = 1.00
	IBW	0.075	0.019	0.020	− 0.004	0.024	0.032	0.294*
								(0.26 to 0.32)
								Pr(h^2^ > 0) = 1.00

Estimates for piglet survival at birth (SVB), during the nursing period (SVNP) and individual piglet birth weight (IBW) are presented as posterior means of genetic variances, direct and maternal heritabilities h^2^ (on the diagonal), genetic correlations ${\text{r}}_{\text{g}}$ (above the diagonal) including their 95% highest posterior density interval (in parentheses), posterior probability of being positive Pr(. > 0) or negative Pr(. < 0) and genetic covariances (below the diagonal) using a Bayesian multivariate analysis at the piglet level

*Significantly different from 0 (P < 0.05)

#### Litter and residual environmental effect

Estimates of the phenotypic proportions of common environmental variances within litter at the piglet level for SVB, SVNP, and IBW were significant and ranged from 0.08 to 0.15 (Table 3). The maternal genetic and litter variances explained cumulatively 0.19 to 0.42 of the phenotypic variance for the analysed traits. Estimates of correlations between litter effects for SVB, SVNP, and IBW were positive but low, and estimates of residual environmental correlations were moderately high and positive.

**Table 3:** Estimated parameters for litter effects for piglet survival traits and birth weight and their residual environmental correlations at the piglet level

Trait	SVB	SVNP	IBW
SVB	0.153	0.368	0.218
	(0.11 to 0.19)	(0.20 to 0.54)	(0.12 to 0.32)
	Pr(l^2^ > 0) = 100	Pr(r_l_ > 0) = 1.00	Pr(r_l_ > 0) = 1.00
SVNP	0.565	0.076	0.361
	(0.32 to 0.81)	(0.06 to 0.09)	(0.27 to 0.45)
	Pr(r_e_ > 0) = 1.00	Pr(l^2^ > 0) = 100	Pr(r_l_ > 0) = 1.00
IBW	0.412	0.465	0.121
	(0.35 to 0.47)	(0.42 to 0.51)	(0.11 to 0.13)
	Pr(r_e_ > 0) = 1.00	Pr(l^2^ > 0) = 100	Pr(l^2^ > 0) = 100

Estimated phenotypic proportions of litter effects l^2^ (on diagonal), correlations between litter effects ${\text{r}}_{\text{l}}$ (above the diagonal), residual environmental correlation ${\text{r}}_{\text{e}}$, (below the diagonal) including their 95% highest posterior density interval (in parentheses), posterior probability of being positive Pr(. > 0) for piglet survival traits and birth weight using a Bayesian multivariate analysis at the piglet level

### Variance components at the sow level

Estimates of genetic parameter at the sow level based on a Bayesian multivariate analysis of NB, ABW, SDBW, SVLB, and SVLNP are in Table 4. All heritability estimates were significantly different from 0, as indicated by the lower limit of the 95-HPD interval, which is always above 0. The estimate of heritability for NB was almost twice as high as that for postnatal piglet survival. Surprisingly, the estimate of heritability of ABW was as low as that of NB. The estimate of heritability for variation of birth weight within litter was only half of that of ABW.

**Table 4:** Estimated genetic parameters for piglet survival and birth weight traits analysed at the sow level

Trait	Genetic variance	NB	ABW	SDBW	SVLB	SVLNP
NB	1.920	0.22	− 0.39	0.38	− 0.37	− 0.36
		(0.16 to 0.30)	(− 0.59 to − 0.17)	(0.12 to 0.65)	(− 0.84 to 0.05)	(− 0.68 to − 0.05)
		Pr(h^2^ > 0) = 1.00	Pr(r_g_ < 0) = 1.00	Pr(r_g_ > 0) = 0.99	Pr(r_g_ < 0) = 0.94	Pr(r_g_ < 0) = 0.98
ABW	0.013	− 0.062	0.22	0.68	− 0.17	0.31
			(0.13 to 0.32)	(0.48 to 0.85)	(− 0.64 to 0.29)	(− 0.04 to 0.65)
			Pr(h^2^ > 0) = 1.00	Pr(r_g_ > 0) = 1.00	Pr(r_g_ < 0) = 0.77	Pr(r_g_ > 0) = 0.95
SDBW	0.001	0.012	0.002	0.11	− 0.43	− 0.02
				(0.06 to 0.17)	(− 0.86 to 0.04)	(− 0.41 to 0.35)
				Pr(h^2^ > 0) = 1.00	Pr(r_g_ < 0) = 0.95	Pr(r_g_ < 0) = 053
SVLB	0.0003	− 0.009	− 0.0003	− 0.0002	0.06	0.18
					(0.02 to 0.12)	(− 0.31 to 0.68)
					Pr(h^2^ > 0) = 1.00	Pr(r_g_ > 0) = 0.76
SVLNP	0.002	− 0.020	0.001	− 0.00001	0.0001	0.13
						(0.06 to 0.19)
						Pr(h^2^ > 0) = 1.00

Estimates of number piglets born (NB), average piglet birth weight per litter (ABW), standard deviation of piglet birth weight within litter (SDBW), piglet survival rate per litter at birth (SVLB) and piglet survival rate per litter during the nursing period (SVLNP) presented as posterior means of genetic variances, direct heritabilities (on the diagonal), genetic correlations ${\text{r}}_{\text{g}}$ (above diagonal) including their 95% highest posterior density interval (in parentheses), posterior probability of being positive Pr(. > 0) or negative Pr(. < 0) and genetic covariances (below the diagonal) using a Bayesian multivariate analysis at the sow level

Estimates of genetic correlations of NB with ABW, SVLB, and SVLNP were all significantly negative, i.e. close to − 0.40, while the estimate of the genetic correlation of NB with SDBW was positive and of similar magnitude, i.e. 0.38. The ABW was estimated to be significantly genetically correlated with SDBW, but not with SVLB and SVLNP. Estimates of the genetic correlation of SDBW with survival traits were negative but non-significant, and only that with SVLB reached a moderate value that approached significance. Estimates of genetic correlations between survival traits were low and positive but not significant.

#### Permanent environmental and residual effects

Estimates of the phenotypic proportions of permanent environmental variance of the sow reproduction traits are summarised in Table 5. The permanent environmental effects for NB and survival traits were small and ranged from 0.04 to 0.09, whereas those for birth weight traits were larger, i.e. from 0.15 to 0.21. Estimates of permanent environmental correlations between traits were not significantly different from 0, except for the negative and moderate correlation for NB with ABW and the high correlation for NB with SDBW. In contrast to the permanent environmental correlations, estimates of correlations between residual effects were all significantly different from zero and ranged from − 0.56 for NB with ABW to 0.28 for ABW with SVLNP.

**Table 5:** Estimated parameters of permanent environmental effects for litter piglet survival and birth weight traits and their residual correlations at the sow level

Trait	NB	ABW	SDBW	SVLB	SVLNP
NB	0.04	− 0.43	− 0.93	0.44	0.09
	(0.02 to 0.06)	(− 0.79 to − 0.04)	(− 1.00 to − 0.84)	(− 0.07 to 0.88)	(− 0.42 to 0.62)
	Pr(pe^2^ > 0) = 100	Pr(r_pe_ < 0) = 0.973	Pr(r_pe_ < 0) = 1.00	Pr(r_pe_ > 0) = 0.94	Pr(r_pe_ > 0) = 0.63
ABW	− 0.56	0.15	0.27	− 0.15	0.09
	(− 0.60 to − 0.52)	(0.06 to 0.24)	(− 0.15 to 0.67)	(− 0.77 to 0.46)	(− 0.49 to 0.65)
	Pr(r_e_ < 0) = 1.00	Pr(pe^2^ > 0) = 100	Pr(r_pe_ > 0) = 0.90	Pr(r_pe_ < 0) = 0.69	Pr(r_pe_ > 0) = 0.64
SDBW	0.12	− 0.28	0.21	− 0.19	− 0.07
	(0.06 to 0.18)	(− 0.34 to − 0.22)	(0.09 to 0.34)	(− 0.76 to 0.34)	(− 0.57 to 0.46)
	Pr(r_e_ > 0) = 1.00	Pr(r_e_ < 0) = 1.00	Pr(pe^2^ > 0) = 100	Pr(r_pe_ < 0) = 0.75	Pr(r_pe_ < 0) = 0.60
SVLB	− 0.15	0.19	− 0.11	0.06	0.29
	(− 0.22 to − 0.09)	(0.13 to 0.26)	(− 0.17 to − 0.05)	(0.02 to 0.11)	(− 0.26 to 0.78)
	Pr(r_e_ < 0) = 1.00	Pr(r_e_ > 0) = 1.00	Pr(r_e_ < 0) = 1.00	Pr(pe^2^ > 0) = 100	Pr(r_pe_ > 0) = 0.85
SVLNP	− 0.21	0.28	− 0.15	0.05	0.09
	(− 0.27 to − 0.15)	(0.22 to 0.34)	(− 0.21 to − 0.09)	(− 0.02 to 0.11)	(0.03 to 0.15)
	Pr(r_e_ < 0) = 1.00	Pr(r_e_ > 0) = 1.00	Pr(r_e_ < 0) = 1.00	Pr(r_e_ > 0) = 0.92	Pr(pe^2^ > 0) = 100

Estimated phenotypic proportions of permanent effects pe^2^ (on diagonal), correlations among permanent effects r_pe_ (above the diagonal), residual correlation r_e_ (below the diagonal), including their 95% highest posterior density interval (in parentheses) and posterior probability of being positive Pr(. > 0) or negative Pr(. < 0) for sow productivity traits using a Bayesian multivariate analysis at the sow level

### Responses to selection

#### Analysis at the piglet level

**Fig. 1:**
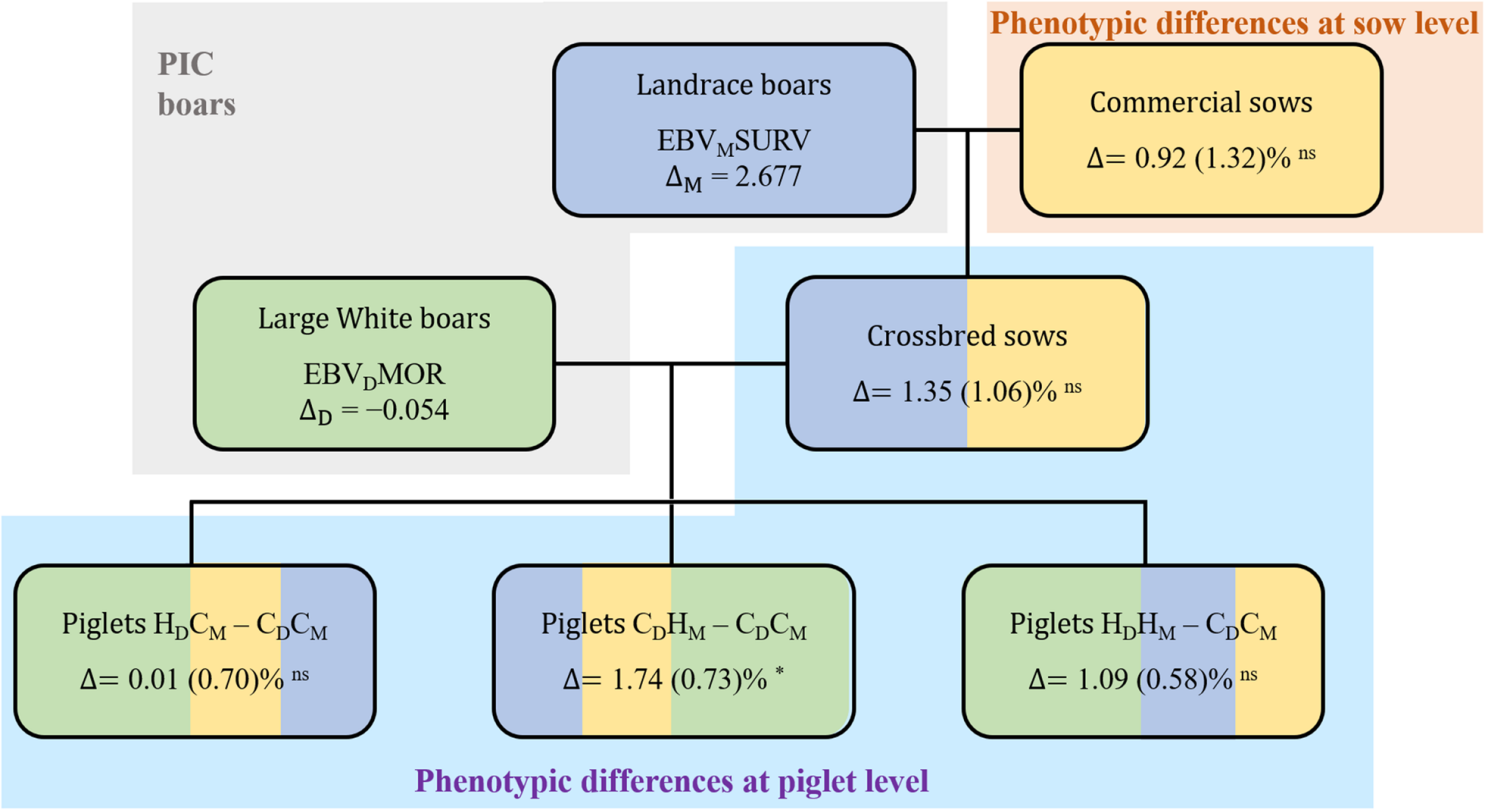
Differences in estimated breeding values between selected and control boars at mating and phenotypic selection responses of piglet survival during the nursing period estimated at the sow and piglet levels. Estimated breeding values (EBV) of Landrace and Large White boars at the time of mating were based on survival (SURV) and mortality (MOR) for maternal and direct effects, respectively. ${\text{H}}$ and ${\text{C}}$ represent high and control breeding groups and subscripts ${\text{D}}$ and ${\text{M}}$ denote direct and maternal genetic effects; *: significantly different from 0 (P < 0.05), ns: non-significant

Figure 1 shows the differences in mean EBV between the high and control groups of Landrace (first generation) and Large White (second generation) boars, which were based on postnatal piglet survival in an indoor production system at PIC, and the mean phenotypic differences between piglets from the different selection groups. For interpretation of differences in EBV between the selection and control groups for postnatal piglet survival, it should be noted that positive maternal and negative direct EBV are desirable because PIC estimated the former based on survival and the latter based on mortality. The highest phenotypic response in postnatal survival was achieved when selection was based on maternal genetic effects only and was 60% higher than the response resulting from tandem selection on both maternal and direct effects. In contrast, no phenotypic response was achieved when selection was on direct genetic effects only. The correlated phenotypic selection responses for SVB and IBW are in Additional file 2: Figure S1 and Additional file 3: Figure S2. They showed consistent improvement of these traits after selection on postnatal survival, but they were significant only for SVB and IBW when selection was on direct genetic effects only and also for IBW when selection was on maternal genetic effects only. Interestingly, selection on both direct and maternal effects of postnatal survival resulted in the lowest and non-significant correlated responses in SVB and IBW.

**Fig. 2:**
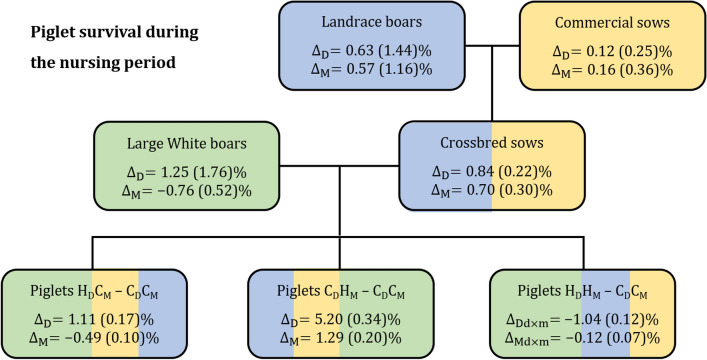
Differences in estimated breeding values of piglet survival during the nursing period between selected (${\mathbf{H}}$) and control (${\mathbf{C}}$) boars based on performances realised within the selection experiment and the selection responses in direct (${\mathbf{D}}$) and maternal (${\mathbf{M}}$) effects of the same trait in crossbred sows and in third-generation piglets originating from different selection scenarios for direct (${\mathbf{H}}_{{\mathbf{D}}} {\mathbf{C}}_{{\mathbf{M}}} - {\mathbf{C}}_{{\mathbf{D}}} {\mathbf{C}}_{{\mathbf{M}}}$) or maternal (${\mathbf{C}}_{{\mathbf{D}}} {\mathbf{H}}_{{\mathbf{M}}} - {\mathbf{C}}_{{\mathbf{D}}} {\mathbf{C}}_{{\mathbf{M}}}$) effects only or their combination (${\mathbf{H}}_{{\mathbf{D}}} {\mathbf{H}}_{{\mathbf{M}}} - {\mathbf{C}}_{{\mathbf{D}}} {\mathbf{C}}_{{\mathbf{M}}}$)

Figure 2 illustrates the estimates of selection responses between the selection and control groups obtained based on estimated direct and maternal breeding values of the third-generation piglets for postnatal piglet survival, separately for each selection scenario. Differences in EBV between the groups of Landrace and Large White boars and first- and second-generation sows based on piglet survival performance obtained within the experiment are also presented. Selection on maternal genetic effects only, represented by its deviation from the control ${\text{C}}_{{\text{D}}} {{\text{H}}}_{{\text{M}}} - {{\text{C}}}_{{\text{D}}} {{\text{C}}}_{\text{M}}$, led to a significant increase in maternal response of 1.3% in postnatal piglet survival. Surprisingly, the correlated direct genetic response in postnatal survival was even higher, at 5.2%, although no selection on direct genetic effects was performed. Selection on direct genetic effects only (${\text{H}}_{{\text{D}}} {{\text{C}}}_{{\text{M}}} - {{\text{C}}}_{{\text{D}}} {{\text{C}}}_{{\text{M}}}$) resulted in a direct response of 1.1% but was associated with a high negative correlated response of − 0.5% in maternal genetic effects of SVNP, so that the overall response was only 0.6%. Selection on both direct and maternal genetic effects (${\text{H}}_{{\text{D}}} {{\text{H}}}_{{\text{M}}} - {{\text{C}}}_{{\text{D}}} {{\text{C}}}_{{\text{M}}}$) resulted in a 1% lower direct response compared to its expected response derived in Eq. (3) and a 0.1% lower maternal response than its expected response derived in Eq. (6). This indicates a substantial reduction in direct response when selection was on both direct and maternal genetic effects, while the maternal response was not significantly affected. Although Landrace boars were selected only on maternal EBV provided by PIC, based on indoor SVNP performance, their EBV for the same trait recorded within the experiment in an outdoor production system, were even slightly higher for direct effects than for maternal effects. However, these differences can only be interpreted as a tendency because of the small number of boars used. For Large White boars, which were selected on direct EBV for SVNP based on indoor reared pigs, the differences in average direct EBV between the selection and control groups were positive, i.e. 1.3%, but negative, i.e. − 0.8%, for maternal genetic effects. These differences should also be interpreted only as a tendency because of their large standard errors. For the commercial sows used in the first-generation, very small differences in average direct and maternal EBV were observed between the selection and control groups, resulting in a negligible impact on the selection response achieved in the third-generation piglets. For the second-generation crossbred sows and the Landrace sires, positive direct and maternal selection responses in SVNP were observed, although the estimate of the genetic correlation between these effects was negative i.e. − 0.15, and selection was on maternal genetic effects only.

**Fig. 3:**
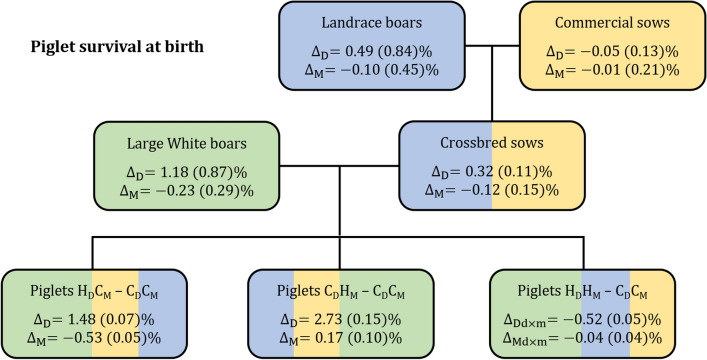
Differences in estimated breeding values of piglet survival at birth of boars selected on postnatal survival and the correlated selection responses estimated as differences between the high (${\mathbf{H}}$) and control (${\mathbf{C}}$) groups in direct (${\mathbf{D}}$) and maternal (${\mathbf{M}}$) effects of the survival at birth in crossbred sows and in third-generation piglets originating from different selection scenarios for direct (${\mathbf{H}}_{{\mathbf{D}}} {\mathbf{C}}_{{\mathbf{M}}} - {\mathbf{C}}_{{\mathbf{D}}} {\mathbf{C}}_{{\mathbf{M}}}$) or maternal (${\mathbf{C}}_{{\mathbf{D}}} {\mathbf{H}}_{{\mathbf{M}}} - {\mathbf{C}}_{{\mathbf{D}}} {\mathbf{C}}_{{\mathbf{M}}}$) effects only or their combination (${\mathbf{H}}_{{\mathbf{D}}} {\mathbf{H}}_{{\mathbf{M}}} - {\mathbf{C}}_{{\mathbf{D}}} {\mathbf{C}}_{{\mathbf{M}}}$)

Selection on SVNP resulted in a significant positive correlated response for SVB for direct genetic effects only, even when selection was on maternal genetic effects for SVNP (Fig. 3). When selection for SVNP was on direct genetic effects only, the correlated selection responses in maternal genetic effects for SVB were negative, i.e. − 0.5%, such that the overall correlated response was reduced to 1.0%. Selection on both direct and maternal effects reduced the direct response significantly but not the maternal response compared to their expectations based on single-effect selection. An unexpected result was the correlated positive direct response of the second-generation crossbred sows for SVB because their sires were selected on maternal genetic effects for SVNP and the estimate of the direct-maternal genetic correlation for SVB was not significant, i.e. 0.15.

**Fig. 4:**
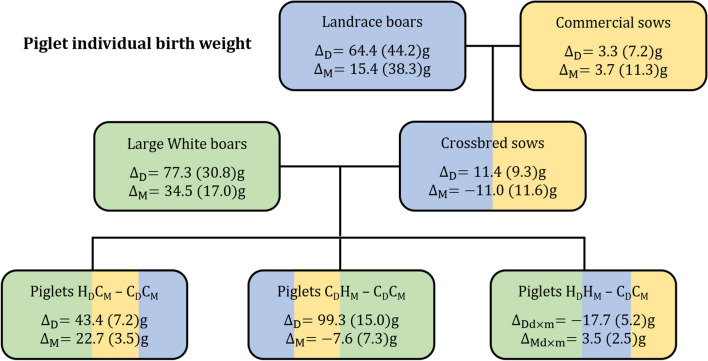
Differences in estimated breeding values of piglet individual birth weight of boars selected on postnatal survival and the correlated selection responses estimated as differences between the high (${\mathbf{H}}$) and control (${\mathbf{C}}$) in direct (${\mathbf{D}}$) and maternal (${\mathbf{M}}$) effects of birth weight in crossbred sows and in third-generation piglets originating from different selection scenarios for direct (${\mathbf{H}}_{{\mathbf{D}}} {\mathbf{C}}_{{\mathbf{M}}} - {\mathbf{C}}_{{\mathbf{D}}} {\mathbf{C}}_{{\mathbf{M}}}$) or maternal (${\mathbf{C}}_{{\mathbf{D}}} {\mathbf{H}}_{{\mathbf{M}}} - {\mathbf{C}}_{{\mathbf{D}}} {\mathbf{C}}_{{\mathbf{M}}}$) effects only or their combination (${\mathbf{H}}_{{\mathbf{D}}} {\mathbf{H}}_{{\mathbf{M}}} - {\mathbf{C}}_{{\mathbf{D}}} {\mathbf{C}}_{{\mathbf{M}}}$)

As for SVB, positive correlated responses of 43 and 99 g were achieved for direct genetic effects of IBW when selection was on direct or maternal effects for postnatal survival, respectively (Fig. 4). However, in contrast to SVB, a positive maternal response of 23 g was also found for IBW when selection was on direct genetic effects only. Consistent with the results for survival traits, a reduction in birth weight was observed when selection was on both direct and maternal effects. The averages for the selection and control groups, on which the direct and maternal responses of survival traits and IBW were based, are in Additional file 4: Table S2 and Additional file 5: Table S4.

Adjustment of piglet survival traits and individual birth weight for litter size resulted in a reduction of direct and maternal selection response for postnatal survival from 1.1 to 0.5% and from 1.2 to 0.8%, respectively (see Additional file 4: Table S3 and Additional file 5: Table S5).

#### Analysis at the sow level

Selection for postnatal survival resulted in a significant maternal selection response of 2.6% in SVLNP in the second-generation crossbred sows (Table 6). Significant correlated responses of − 0.68 piglets and − 9 g were obtained in NB and SDBW, respectively. Tendencies of positively correlated responses of 5 g in ABW and of 0.2% in SVLB were also observed but these were not significantly different from 0.

**Table 6:** Selection response for sow productivity traits

Trait	Group	LSM	SE	*P* value
Number piglets born (piglet/litter)	${\text{C}}_{\text{M}}$	0.458	0.050	< 0.0001
	${\text{H}}_{\text{M}}$	0.116	0.049	0.027
	${\text{R}} = 2\left( {{\text{H}}_{\text{M}} {-}{\text{C}}_{\text{M}} } \right)$	− 0.684	0.140	< 0.0001
Average piglet birth weight per litter (kg)	${\text{C}}_{\text{M}}$	− 0.025	0.005	0.0000
	${\text{H}}_{\text{M}}$	− 0.023	0.005	0.0000
	${\text{R}} = 2\left( {{\text{H}}_{\text{M}} {-}{\text{C}}_{\text{M}} } \right)$	0.005	0.014	0.735
Standard deviation of piglet birth weight within litter (kg)	${\text{C}}_{\text{M}}$	0.001	0.001	0.273
	${\text{H}}_{\text{M}}$	− 0.003	0.001	0.0001
	${\text{R}} = 2\left( {{\text{H}}_{\text{M}} {-}{\text{C}}_{\text{M}} } \right)$	− 0.009	0.003	0.0007
Piglet survival rate per litter at birth (%/litter)	${\text{C}}_{\text{M}}$	− 0.089	0.041	0.026
	${\text{H}}_{\text{M}}$	0.009	0.040	0.833
	${\text{R}} = 2\left( {{\text{H}}_{\text{M}} {-}{\text{C}}_{\text{M}} } \right)$	0.195	0.114	0.087
Piglet survival rate per litter during the nursing period (%/litter)	${\text{C}}_{\text{M}}$	− 1.023	0.113	< 0.0001
	${\text{H}}_{\text{M}}$	0.285	0.112	0.007
	${\text{R}} = 2\left( {{\text{H}}_{\text{M}} {-}{\text{C}}_{\text{M}} } \right)$	2.616	0.319	< 0.0001

Least squares mean (LSM) of breeding values for the high and control groups (${\text{H}}_{\text{M}}$ and ${\text{C}}_{\text{M}}$) and their comparisons to be used to estimate the selection response R for various sow productivity traits. SE, standard error

## Discussion

We found averages for number of piglets born alive, survival percentage at birth per litter and survival percentage during the nursing period of 12.3 piglets, and 96.7 and 89%, respectively. The averages for performances reported in 2007 were slightly smaller for litter size but higher for survival traits (12.9 piglets, and 94.9 and 87.6%) compared to the average 2018 performance on British farms [7].

### Variance components at the piglet level

A comprehensive discussion on genetic parameters for piglet survival and birth weight traits at the piglet level using the same data was presented by Roehe et al. [24]. In the current study, we used an updated software and a longer Gibbs chain and found that the antagonistic correlation between direct and maternal effects within traits was less strong compared to the estimate of Roehe et al. [24] and that the reduction in marginal posterior distributions of some low estimates of correlations between direct and maternal effects of different traits resulted in their significance, which indicates that using a large number of Gibbs sampling iterations and a longer burn-in period reduced the HPD interval of genetic parameters, in particular when using a complex linear and threshold model with direct and maternal effects.

For interpretation of the selection response, it is important to note that estimates of the direct or maternal genetic correlations between survival traits were significantly positive at ~ 0.2. This low genetic correlation indicates that the survival traits should be considered as different traits when estimating response to selection. Estimates of genetic correlations between survival traits and birth weight were also significantly different from zero and low, thus an increased birth weight is expected to improve piglet survival only slightly. However, the relationships of birth weight with survival traits are non-linear at the phenotypic level [see Additional file 6: Figures S3 and S4], and this is not be reflected by the estimated linear genetic correlations. Furthermore, adjustment of the piglet traits for litter size affected their genetic parameter estimates only marginally, which could be because the impact of litter size on piglet survival and birth weight was indirectly accounted for in the model by the common environmental litter effect.

### Variance components at the sow level

The estimate of the heritability of NB was higher than the estimates reported in [29–32], which ranged from 0.12 to 0.16, whereas the estimates of heritability of SVLB and SVLNP were in agreement with those found in the literature, which ranged from 0.04 to 0.20 [17, 20, 33]. For birth weight traits at the sow level, estimates of heritability were of the same order of magnitude as most of the estimates reported in the literature, which ranged from 0.11 to 0.27 for SDBW [17, 31, 34] and from 0.19 to 0.34 for ABW [17, 32–35], but were lower than the estimate reported by Banville et al. [31] for the same trait (0.51).

The negative estimates of genetic correlations of NB with survival traits and ABW indicate an antagonism between litter size and, piglet survival and birth weight, which confirm the negative correlations of NB with SVLB and SVLNP that were reported by Su et al. [9] and Matheson et al. [35] (ranging from − 0.07 to − 0.52), and the positive genetic correlation of NB with piglet mortality that was reported by Putz et al. [36] (0.23).

Damgaard et al. [37] and Wolf et al. [38] reported a positive genetic correlation between within-litter birth weight variation and piglet mortality, which agrees with the negative genetic correlations of SDBW with SVLNP and SVLB found here, but which were not significant. Further analyses using canalized selection are needed to understand the genetic relationships of variability of piglet birth weight with survivability of piglets [39–41].

### Selection responses

In the experiment used for this study, selection on maternal or direct genetic effects of postnatal piglet survival only, successfully achieved an increase of more than 1% in survival for each genetic effect under selection. The substantial selection responses obtained also revealed that there are no strong genotype-by-environment interactions between survival of piglets reared under indoor or outdoor conditions because the EBV for selection were obtained based on postnatal piglet survival recorded indoors, whereas data recorded in the selection experiment were collected outdoors. In addition, since the boars were not selected for both extremes but for high and average EBV of postnatal survival, the achieved selection response has to be considered as high. The boars used as the control group had EBV for postnatal survival (provided by PIC) close to zero, with the aim to produce a control population unselected for postnatal survival.

A surprising result was that the selection response in the scenario in which only maternal genetic effects were selected for produced a four-times higher response in direct than maternal genetic effects. This is most likely due to unintentional selection of the best piglets within a litter when choosing gilts for use as the second-generation crossbred sows, which may, as a result, be genetically superior for growth rate, vitality, conformation, health, etc. This is supported by the high estimated direct response in these crossbred second-generation sows, although they were sired by Landrace boars that were selected for high maternal breeding values only, and a negative correlation between direct and maternal effects was used for estimation of breeding values. This unintentional selection is also supported by the higher direct EBV for IBW of the crossbred sows. Moreover, phenotypically, the chosen gilts were on average 104 ± 24 g heavier at birth than the non-selected gilts. Because the high direct response due to unintentional selection only occurred in the high maternal selection group, it suggests that a high potential for maternal genetic effects is necessary to achieve substantial direct genetic response in postnatal piglet survival. This agrees with studies that show that piglet survival depends on uterine capacity, foetus placental quality, colostrum production and nutrient transfer from their mothers, and on adaptive immunity [10, 42–45]. In addition, postnatal piglet survival is affected by the sow’s farrowing behaviours, such as lying behaviour and (lower) aggression towards piglets. Andersen et al. [46] observed that sows that did not crush their piglets reacted more quickly to piglet sounds and nosed them more frequently to alert the piglets that were lying down, whereas Baxter et al. [47] found that postnatal piglet mortality of sows with aggressive behaviours, such as pawing, rooting or biting, was elevated. Such maternal genetic effects are the first limiting factor for postnatal survival that we detected in the scenario in which only direct genetic effects were selected for, because the direct genetic response obtained was partly offset by a negative maternal genetic response in that scenario. This was validated by the scenario in which both direct and maternal effects were selected, which resulted in a significant negative direct response as deviation from its expected single-effect’s response. In contrast, in this selection scenario, the deviation of the maternal response from its expected single-effect’s response was not significant. The fact that the maternal effects are a limiting factor for direct response would also explain why the unintentional selection for traits that were correlated with direct genetic effects of postnatal piglet survival, resulted in a high direct response when only maternal effects were selected on because in that case the unintentional selection was less restricted by maternal effects.

Heterosis could also influence the observed selection responses but Johansson et al. [48] and Roehe et al. [49] showed that most of the non-additive genetic effects are captured by the litter effect, so heterosis most likely did not bias the EBV.

The negative genetic correlation between direct and maternal genetic effects had a substantial impact on the observed direction and magnitude of the selection responses in postnatal survival, in particular when selection was on direct genetic effects. In a previous simulation study, Roehe et al. [50, 51] found that a negative genetic correlation between direct and maternal effects resulted in a substantial reduction in overall response and, as our results from the selection experiment suggest, this is even the case when this correlation is small, as it is for postnatal survival (-0.15). The biological constraints discussed above, i.e. that the maternal genetic effects are the first limiting factor for improving postnatal survival, may increase the undesirable effect of a negative direct-maternal correlation on response to selection, in particular, if the selection is only on the direct genetic effects of piglet survival.

Another unexpected result was the correlated positive direct response in SVB when selection was on maternal effects for SVNP in the second-generation crossbred sows. Again, this result illustrates the unintentional selection of the best gilts within litters to become the second-generation sows, on traits that were correlated with direct genetic effects, such as growth rate, vitality, and conformation. This unintentional selection had a stronger effect in the high maternal selection group than in the corresponding control group. Similar to the selection response for SVNP, when selection was on direct genetic effects, the correlated response in maternal effects for SVB was high and negative. Again, this indicates the antagonistic correlation between direct and maternal effects of piglet survival, in particular when selection on direct effects is in a population with limited maternal resources.

Correlated responses in IBW from selection only on direct or maternal effects for SVNP were all positive for direct effects and substantially higher than correlated responses for maternal effects, which suggest that selection for postnatal survival acted on the piglet genes associated with growth, in particular. Generally, the correlated response observed for IBW suggests that there is a positive genetic correlation between postnatal survival and piglet growth. However, a reduction in direct response in intrauterine growth compared to its expectation has to be considered when selection is on both direct and maternal effects for postnatal piglet survival. The antagonistic selection responses for direct *versus* maternal effects of the analysed piglet traits can be explained only partly by the low estimates of genetic correlations in the population. High selection pressure on direct effects for piglet postnatal survival substantially increased the antagonism. Thus, our results emphasize that the genetics that control the maternal traits of sows, such as the provision of milk and mothering abilities, might not be sufficient to support the potential postnatal survival with selection on direct genetic effects only. Therefore, optimal selection on direct and maternal effects needs to emphasize maternal genetic effects of piglet survival.

The estimated phenotypic responses to selection were consistent with the estimated genetic responses, considering that only one quarter and one half of the responses to the first- and second-generation selection are expressed phenotypically, as derived in Eqs. (1) to (6). The expected phenotypic responses in postnatal survival based on the estimated genetic responses would be 0.31, 1.62 and 0.77% when selection was on direct or maternal effects only or their combination, respectively. These values are close to the phenotypic responses in Fig. 1, in particular when considering the standard errors in the phenotypic responses. The impact of litter size on the genetic response was identified after phenotypic adjustment of this factor in the model at the piglet level, which resulted in reductions in direct and maternal responses in SVNP by 56 and 34%, respectively, when selection was only on the corresponding genetic effects. This shows that the EBV and thus selection responses in SVNP did substantially change due to adjustment for litter size, although the genetic parameters were only to a negligible extent affected by this adjustment. However, this is a phenotypic adjustment of postnatal piglet survival for litter size on the piglet level, while the analyses of survival and litter size traits at the sow level are providing the genetic correlations and correlated response in those traits due to selection for postnatal piglet survival.

At the sow level, a substantial improvement in postnatal piglet survival (2.6%) was achieved in the selection experiment. However, there was a trade-off of 0.68 less piglets born in the selection group than in the control group. Considering the high costs of losing a piglet postnatally, the additional feed costs, and the ethical and animal welfare aspects, we believe that the higher piglet survival more than offsets the smaller number of piglets born. Based on their estimated negative genetic correlation (− 0.36), the impact of selection for postnatal piglet survival on litter size was expected, and agreed with other studies [9, 52]. Hill et al. [53] suggested one solution to overcome this negative effect by stabilizing and directional selection on environmental variation using canalized selection. This opens up the opportunity to select for an optimal level of litter size and birth weight, while continuing to improve piglet survival [41, 54–56].

## Conclusions

The results obtained from this two-generation selection experiment demonstrate that selection for piglet survival can be highly successful, in particular when the selection is on maternal genetic effects only. In particular, selection on direct effects, but to a lesser extent also on both direct and maternal effects, substantially increased the antagonism between direct and maternal genetic effects for piglet survival and thus reduced its overall genetic response due to unbalanced breeding for these genetic effects. Biologically, this suggests that maternal effects such as milk yield, mothering ability, etc. are the first limiting factor for selection response in piglet survival and highlights the importance of maternal selection to improve piglet survival. A higher phenotypic birth weight of second-generation selected sows suggests that unintentional selection of the best gilts within litters most likely based on growth, vitality, and conformation increased the direct genetic response in postnatal survival, especially when selection was on maternal genetic effects only. This demonstrated that the direct genetic response was expressed only when it was not limited by maternal genetic effects. In practical breeding, this unintentional selection could be considered in the breeding evaluation by including these traits in a multivariate analysis with piglet survival. Furthermore, the increase in piglet birth weight due to selection on maternal or direct genetic effects of postnatal piglet survival suggests that an improvement of birth weight is necessary to enhance piglet survival. A drawback of selecting boars based only on their maternal and direct EBV for postnatal survival was a correlated negative response in litter size of − 0.68 piglets, which highlights the necessity to select simultaneously for both postnatal survival and litter size. Thus, the use of canalised selection for stabilizing and directional selection for litter size and birth weight might improve piglet survival by exploiting the limited maternal resources more efficiently. Because the boars used in the experiment were selected based on postnatal survival of piglets produced indoors, while the selection experiment was carried out under outdoor rearing, the selection response obtained in postnatal piglet survival suggests that genotype-by-environment interactions between piglet survival under indoor and outdoor conditions were not important.

## Supplementary Information

**Additional file 1: Table S1.** Estimated genetic parameters for piglet survival traits and birth weight analysed at the piglet level after adjustment of these traits for litter size. Estimates for piglet survival at birth (SVB), during the nursing period (SVNP) and individual piglet birth weight (IBW) are presented as posterior means of direct and maternal heritabilities h^2^ (on the diagonal), genetic correlations r_g_ (above the diagonal) including their 95% highest posterior density interval (in parentheses), posterior probability of being positive Pr(. > 0) or negative Pr(. < 0) and genetic covariances (below diagonal) using a Bayesian multivariate analysis at the piglet level; *: significantly different from 0 (P < 0.05).

**Additional file 2: Figure S1.** Correlated phenotypic responses of piglet survival at birth due to selection for postnatal survival estimated at the sow and piglet levels. Summary of phenotypic responses of piglet survival at birth in three selection scenarios based on piglet level in the 2^nd^ and 3^rd^ generation, along with phenotypic differences in piglet survival rate per litter at birth of sows in 1^st^ generation.

**Additional file 3: Figure S2.** Correlated phenotypic responses of piglet individual birth weight due to selection for postnatal survival estimated at the sow and piglet levels. Summary of phenotypic responses of piglet individual birthweight in three different selection scenarios of piglets in 2^nd^ and 3^nd^ generation, along with phenotypic differences in average piglet birth weight per litter of sows in 1^st^ population.

**Additional file 4: Table S2.** Maternal selection responses in survival traits and birth weight at the piglet level. Summary of maternal selection responses of three different selection scenarios of selection for postnatal piglet survival and their correlated responses in perinatal survival and individual birth weight. **Table S3.** Maternal selection responses in survival traits and birth weight at the piglet level after adjustment of these traits for litter size. Summary of maternal selection responses of three different selection scenarios of selection for postnatal piglet survival and their correlated responses in perinatal survival and individual birth weight after adjustment of these traits for litter size.

**Additional file 5: Table S4**. Direct selection responses in survival traits and birth weight at the piglet level. Summary of direct selection responses of three different selection scenarios of selection for postnatal piglet survival and their correlated responses in perinatal survival and individual birth weight. **Table S5.** Direct selection responses in survival traits and birth weight at the piglet level after adjustment of these traits for litter size. Summary of direct selection responses of three different selection scenarios of selection for postnatal piglet survival and their correlated responses in perinatal survival and individual birth weight after adjustment of these traits for litter size.

**Additional file 6: Figure S3.** Generalised linear regression (fitting a probit link function) of survival at birth (SVB) on individual birthweight (IBW), including confidence interval at 95%. The figure shows the generalised linear regression (fitting a probit link function) of survival at birth (SVB) on individual birthweight (IBW), including confidence interval at 95%. **Figure S4.** Generalised linear regression (fitting a probit link function) of survival at birth (SVB) on individual birthweight (IBW), including confidence interval at 95%. The figure shows the generalised linear regression (fitting a probit link function) of survival during the nursing period (SVNP) on individual birthweight (IBW), including confidence interval at 95%.

## Data Availability

Data are available only upon agreement with the breeding organization and should be requested directly from the authors.
