# Supplementary material for: Evaluation of direct and maternal responses in reproduction traits based on different selection strategies for postnatal piglet survival in a selection experiment
Source: Genet Sel Evol. 2021 Mar 15;53:28. doi: 10.1186/s12711-021-00612-7 (PMC7958901; doi:10.1186/s12711-021-00612-7)
Supplement: Supplementary file 1 — Additional file 1: Table S1. Estimated genetic parameters for piglet survival traits and birth weight analysed at the piglet level after adjustment of these traits for litter size. Estimates for piglet survival at birth (SVB), during the nursing period (SVNP) and individual piglet birth weight (IBW) are presented as posterior means of direct and maternal heritabilities h2 (on the diagonal), genetic correlations rg (above the diagonal) including their 95% highest posterior density interval (in parentheses), posterior probability of being positive Pr(. > 0) or negative Pr(. < 0) and genetic covariances (below diagonal) using a Bayesian multivariate analysis at the piglet level; *: significantly different from 0 (P < 0.05). [file 12711_2021_612_MOESM1_ESM.docx]

**Additional file 1 Table S1 Estimated genetic parameters for piglet survival traits and birth weight analysed at the piglet level after adjustment of these traits for litter size**

| **Effect** | **Trait** | **Genetic variance** | **Direct** | |  | | **Maternal** | | |
| --- | --- | --- | --- | --- | --- | --- | --- | --- | --- |
|  |  |  | **SVB** | **SVNP** | | **IBW** | **SVB** | **SVNP** | **IBW** |
| Direct | SVB | 0.333 | 0.179* | 0.207* | | 0.219* | – 0.052 | 0.137 | 0.090 |
|  |  |  | (0.13 , 0.23) | (-0.02 , 0.43) | | (0.09 , 0.35) | (-0.26 , 0.16) | (-0.06 , 0.33) | (-0.03 , 0.21) |
|  |  |  | Pr(h^2^>0)=1.00 | Pr(r_g_>0)=0.96 | | Pr(r_g_>0)=1.00 | Pr(r_g_<0)=0.68 | Pr(r_g_>0)=0.91 | Pr(r_g_>0)=0.92 |
|  | SVNP | 0.281 | 0.063 | 0.183* | | 0.239* | 0.162 | – 0.169 | 0.112 |
|  |  |  |  | (0.14 , 0.24) | | (0.12 , 0.36) | (-0.04 , 0.36) | (-0.35 , 0.01) | (-0.01 , 0.23) |
|  |  |  |  | Pr(h^2^>0)=1.00 | | Pr(r_g_>0)=1.00 | Pr(r_g_>0)=0.94 | Pr(r_g_<0)=0.97 | Pr(r_g_>0)=0.96 |
|  | IBW | 0.090 | 0.038 | 0.038 | | 0.379* | 0.160* | 0.141* | – 0.077 |
|  |  |  |  |  | | (0.34 , 0.42) | (0.03 , 0.29) | (0.02 , 0.26) | (-0.14 , 0.01) |
|  |  |  |  |  | | Pr(h^2^>0)=1.00 | Pr(r_g_>0)=0.99 | Pr(r_g_>0)=0.99 | Pr(r_g_<0)=0.94 |
| Maternal | SVB | 0.259 | -0.017 | 0.043 | | 0.024 | 0.140* | 0.215* | 0.134* |
|  |  |  |  |  | |  | (0.11 , 0.17) | (0.05 , 0.37) | (0.02 , 0.24) |
|  |  |  |  |  | |  | Pr(h^2^>0)=1.00 | Pr(r_g_>0)=1.00 | Pr(r_g_>0)=1.00 |
|  | SVNP | 0.177 | 0.033 | -0.039 | | 0.018 | 0.046 | 0.116* | 0.242* |
|  |  |  |  |  | |  |  | (0.09 , 0.14) | (0.14 , 0.34) |
|  |  |  |  |  | |  |  | Pr(h^2^>0)=1.00 | Pr(r_g_>0)=1.00 |
|  | IBW | 0.067 | 0.013 | 0.015 | | -0.006 | 0.018 | 0.026 | 0.285* |
|  |  |  |  |  | |  |  |  | (0.26 , 0.31) |
|  |  |  |  |  | |  |  |  | Pr(h^2^>0)=1.00 |

Estimates for piglet survival at birth (SVB), during the nursing period (SVNP) and individual piglet birth weight (IBW) are presented as posterior means of direct and maternal heritabilities h^2^ (on diagonal), genetic correlations r_g_ (above the diagonal) including their 95% highest posterior density interval (in parentheses), posterior probability of being positive Pr(.>0) or negative Pr(.<0) and genetic covariances (below the diagonal) using a Bayesian multivariate analysis at the piglet level

*: significantly different from 0 (P < 0.05).
