# Supplementary material for: Evaluation of direct and maternal responses in reproduction traits based on different selection strategies for postnatal piglet survival in a selection experiment
Source: Genet Sel Evol. 2021 Mar 15;53:28. doi: 10.1186/s12711-021-00612-7 (PMC7958901; doi:10.1186/s12711-021-00612-7)

**Additional file 2 Figure S1 Correlated phenotypic responses of piglet survival at birth due to selection on postnatal survival estimated at the sow and piglet levels.**

H and C represent high and control groups and the subscripts D and M denote direct and maternal genetic effects; * indicate significant response, ns indicate non-significant response.


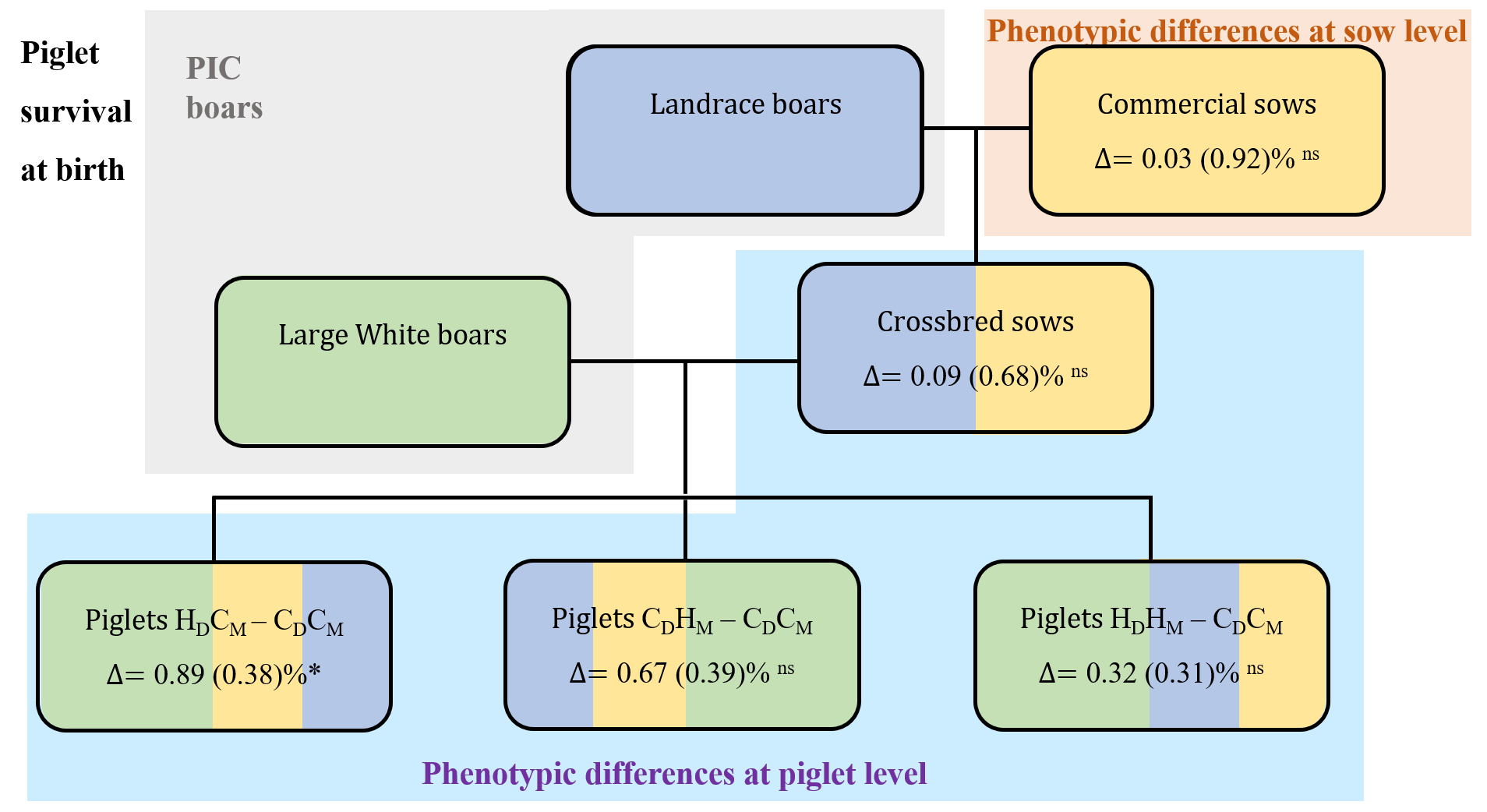

Supplement: Supplementary file 2 — Additional file 2: Figure S1. Correlated phenotypic responses of piglet survival at birth due to selection for postnatal survival estimated at the sow and piglet levels. Summary of phenotypic responses of piglet survival at birth in three selection scenarios based on piglet level in the 2nd and 3rd generation, along with phenotypic differences in piglet survival rate per litter at birth of sows in 1st generation. [file 12711_2021_612_MOESM2_ESM.docx]
