# Supplementary material for: Evaluation of direct and maternal responses in reproduction traits based on different selection strategies for postnatal piglet survival in a selection experiment
Source: Genet Sel Evol. 2021 Mar 15;53:28. doi: 10.1186/s12711-021-00612-7 (PMC7958901; doi:10.1186/s12711-021-00612-7)

**Additional file 3 Figure S2 Correlated phenotypic responses of piglet birth weight due to selection on postnatal survival estimated at the sow and piglet levels**

H and C represent high and control groups and the subscripts D and M denote direct and maternal genetic effects; * indicate significant response, ns indicate non-significant response.


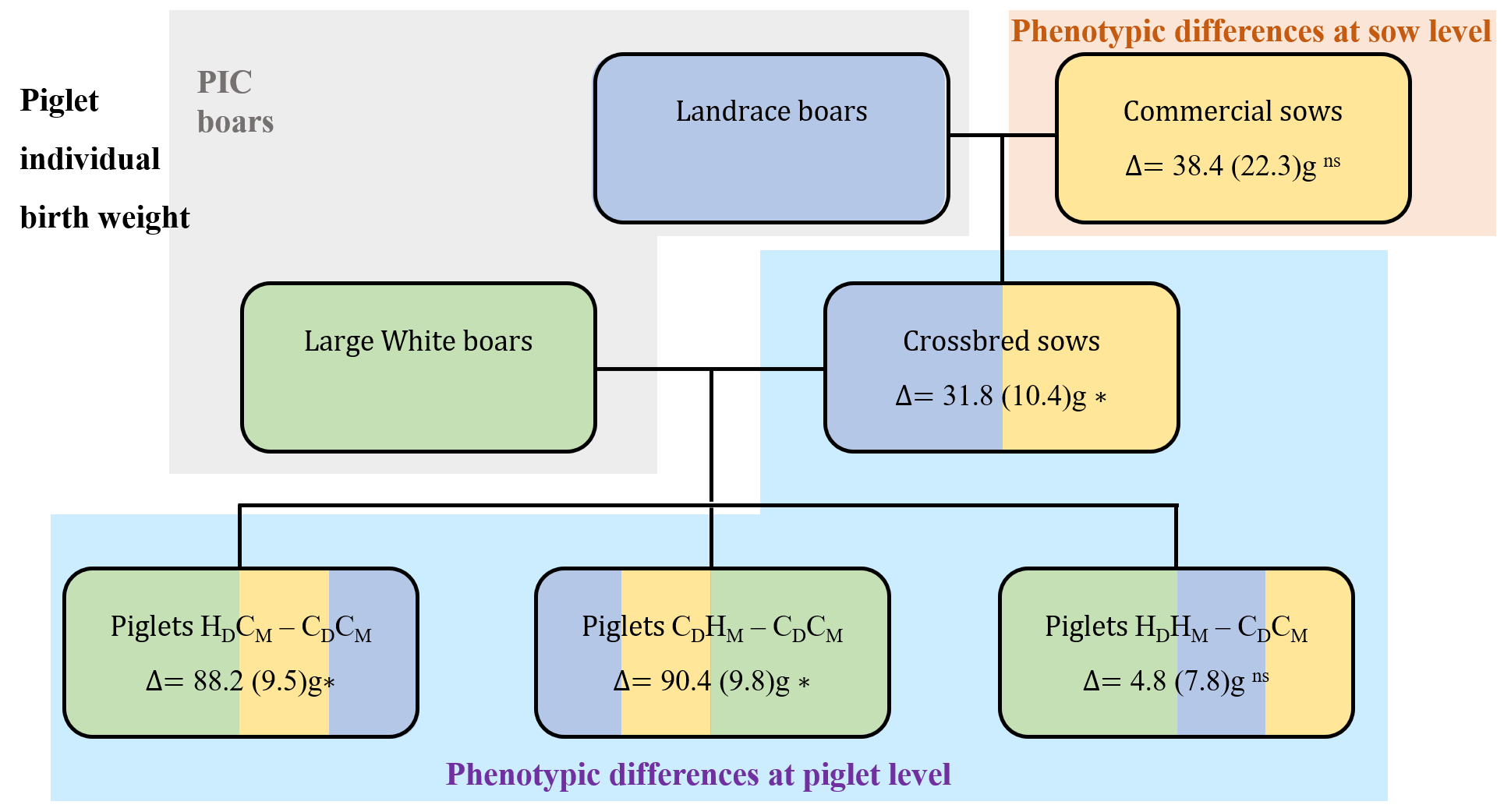

Supplement: Supplementary file 3 — Additional file 3: Figure S2. Correlated phenotypic responses of piglet individual birth weight due to selection for postnatal survival estimated at the sow and piglet levels. Summary of phenotypic responses of piglet individual birthweight in three different selection scenarios of piglets in 2nd and 3nd generation, along with phenotypic differences in average piglet birth weight per litter of sows in 1st population. [file 12711_2021_612_MOESM3_ESM.docx]
