# Supplementary material for: Evaluation of direct and maternal responses in reproduction traits based on different selection strategies for postnatal piglet survival in a selection experiment
Source: Genet Sel Evol. 2021 Mar 15;53:28. doi: 10.1186/s12711-021-00612-7 (PMC7958901; doi:10.1186/s12711-021-00612-7)
Supplement: Supplementary file 4 — Additional file 4: Table S2. Maternal selection responses in survival traits and birth weight at the piglet level. Summary of maternal selection responses of three different selection scenarios of selection for postnatal piglet survival and their correlated responses in perinatal survival and individual birth weight. Table S3. Maternal selection responses in survival traits and birth weight at the piglet level after adjustment of these traits for litter size. Summary of maternal selection responses of three different selection scenarios of selection for postnatal piglet survival and their correlated responses in perinatal survival and individual birth weight after adjustment of these traits for litter size. [file 12711_2021_612_MOESM4_ESM.docx]

**Additional file 4 Table S2 Maternal selection responses in survival traits and birth weight at the piglet level**

| **Effect and trait** | **Selection group** | **LSM** | **SE** | **P-value** |
| --- | --- | --- | --- | --- |
| Maternal breeding value of SVNP | C_D_C_M_ | 88.416 | 0.028 | <0.0001 |
| (%) | C_D_H_M_ | 88.737 | 0.042 | <0.0001 |
|  | H_D_C_M_ | 88.172 | 0.040 | <0.0001 |
|  | H_D_H_M_ | 88.378 | 0.028 | <0.0001 |
| Maternal selection response of SVNP | Group comparison | $\Delta M$ |  |  |
|  | H_D_C_M_ - C_D_C_M_ | -0.486 | 0.098 | <0.0001 |
|  | C_D_H_M_ - C_D_C_M_ | 1.286 | 0.202 | <0.0001 |
|  | ΔM_d×m_ | -0.116 | 0.070 | 0.301 |
| Maternal breeding value of SVB | C_D_C_M_ | 96.268 | 0.014 | <0.0001 |
| (%) | C_D_H_M_ | 96.309 | 0.021 | <0.0001 |
|  | H_D_C_M_ | 96.002 | 0.020 | <0.0001 |
|  | H_D_H_M_ | 96.009 | 0.014 | <0.0001 |
| Maternal selection response of SVB | Group comparison | $\Delta M$ |  |  |
|  | H_D_C_M_ - C_D_C_M_ | -0.531 | 0.049 | <0.0001 |
|  | C_D_H_M_ - C_D_C_M_ | 0.167 | 0.102 | 0.303 |
|  | ΔM_d×m_ | -0.035 | 0.036 | 0.972 |
| Maternal breeding value of IBW | C_D_C_M_ | -12.7 | 1.0 | <0.0001 |
| (g) | C_D_H_M_ | -14.6 | 1.5 | <0.0001 |
|  | H_D_C_M_ | -1.3 | 1.4 | 0.362 |
|  | H_D_H_M_ | 0.3 | 1.0 | 0.742 |
| Maternal selection response of IBW | Group comparison | $\Delta M$ |  |  |
|  | H_D_C_M_ - C_D_C_M_ | 22.7 | 3.5 | <0.0001 |
|  | C_D_H_M_ - C_D_C_M_ | -7.6 | 7.3 | 0.898 |
|  | ΔM_d×m_ | 3.5 | 2.5 | 0.492 |

Least squares mean (LSM) of maternal breeding values for the various selection group and their comparisons used to estimate the selection responses. H and C represent high and control groups and the subscripts D and M denote direct and maternal genetic effects; $\Delta M$ maternal selection response; ΔM_d×m_ are deviations from the expected response based on single effect selection as derived in equation (6);SVB, survival at birth, SVNP, survival during the nursing period, IBW, individual birth weight; SE standard error.

**Additional file 4 Table S3 Maternal selection responses in survival and birth weight at the piglet level after adjustment of these traits for litter size**

| **Effect and trait** | **Selection group** | **LSM** | **SE** | **P-value** |
| --- | --- | --- | --- | --- |
| Maternal breeding value of SVNP | C_D_C_M_ | 88.697 | 0.030 | <0.0001 |
| (%) | C_D_H_M_ | 88.908 | 0.045 | <0.0001 |
|  | H_D_C_M_ | 88.686 | 0.043 | <0.0001 |
|  | H_D_H_M_ | 88.977 | 0.031 | <0.0001 |
| Maternal selection response of SVNP | Group comparison | $\Delta M$ |  |  |
|  | H_D_C_M_ - C_D_C_M_ | -0.022 | 0.105 | 1.000 |
|  | C_D_H_M_ - C_D_C_M_ | 0.843 | 0.218 | <0.0001 |
|  | ΔM_d×m_ | 0.080 | 0.076 | 0.879 |
| Maternal breeding value of SVB | C_D_C_M_ | 96.523 | 0.013 | <0.0001 |
| (%) | C_D_H_M_ | 96.543 | 0.020 | <0.0001 |
|  | H_D_C_M_ | 96.185 | 0.019 | <0.0001 |
|  | H_D_H_M_ | 96.063 | 0.014 | <0.0001 |
| Maternal selection response of SVB | Group comparison | $\Delta M$ |  |  |
|  | H_D_C_M_ - C_D_C_M_ | -0.676 | 0.047 | <0.0001 |
|  | C_D_H_M_ - C_D_C_M_ | 0.078 | 0.096 | 1.000 |
|  | ΔM_d×m_ | -0.142 | 0.034 | <0.0001 |
| Maternal breeding value of IBW | C_D_C_M_ | -14.6 | 0.9 | <0.0001 |
| (g) | C_D_H_M_ | -15.0 | 1.4 | <0.0001 |
|  | H_D_C_M_ | -7.0 | 1.3 | 0.362 |
|  | H_D_H_M_ | -9.3 | 0.9 | 0.742 |
| Maternal selection response of IBW | Group comparison | $\Delta M$ |  |  |
|  | H_D_C_M_ - C_D_C_M_ | 15.3 | 3.2 | <0.0001 |
|  | C_D_H_M_ - C_D_C_M_ | -1.4 | 6.6 | 1.000 |
|  | ΔM_d×m_ | -2.0 | 2.3 | 1.000 |

Least squares mean (LSM) of maternal breeding values for the various selection group and their comparisons used to estimate the selection responses. H and C represent high and control groups and the subscripts D and M denote direct and maternal genetic effects; $\Delta M$ maternal selection response; ΔM_d×m_ are deviations from the expected response based on single effect selection as derived in equation (6);SVB, survival at birth, SVNP, survival during the nursing period, IBW, individual birth weight; SE standard error.
