# Supplementary material for: Evaluation of direct and maternal responses in reproduction traits based on different selection strategies for postnatal piglet survival in a selection experiment
Source: Genet Sel Evol. 2021 Mar 15;53:28. doi: 10.1186/s12711-021-00612-7 (PMC7958901; doi:10.1186/s12711-021-00612-7)
Supplement: Supplementary file 5 — Additional file 5: Table S4. Direct selection responses in survival traits and birth weight at the piglet level. Summary of direct selection responses of three different selection scenarios of selection for postnatal piglet survival and their correlated responses in perinatal survival and individual birth weight. Table S5. Direct selection responses in survival traits and birth weight at the piglet level after adjustment of these traits for litter size. Summary of direct selection responses of three different selection scenarios of selection for postnatal piglet survival and their correlated responses in perinatal survival and individual birth weight after adjustment of these traits for litter size. [file 12711_2021_612_MOESM5_ESM.docx]

**Additional file 5 Table S4 Direct selection responses in survival traits and birth weight at the piglet level**

| **Effect and trait** | **Selection group** | **LSM** | **SE** | **P-value** |
| --- | --- | --- | --- | --- |
| Direct breeding value of SVNP | C_D_C_M_ | 87.810 | 0.048 | <0.0001 |
| (%) | C_D_H_M_ | 89.111 | 0.071 | <0.0001 |
|  | H_D_C_M_ | 88.364 | 0.068 | <0.0001 |
|  | H_D_H_M_ | 88.622 | 0.048 | <0.0001 |
| Direct selection response of SVNP | Group comparison | $\Delta D$ |  |  |
|  | H_D_C_M_ - C_D_C_M_ | 1.107 | 0.165 | <0.0001 |
|  | C_D_H_M_ - C_D_C_M_ | 5.202 | 0.341 | <0.0001 |
|  | ΔD_d×m_ | -1.042 | 0.119 | <0.0001 |
| Direct breeding value of SVB | C_D_C_M_ | 95.471 | 0.021 | <0.0001 |
| (%) | C_D_H_M_ | 96.153 | 0.032 | <0.0001 |
|  | H_D_C_M_ | 96.210 | 0.031 | <0.0001 |
|  | H_D_H_M_ | 96.369 | 0.022 | <0.0001 |
| Direct selection response of SVB | Group comparison | $\Delta D$ |  |  |
|  | H_D_C_M_ - C_D_C_M_ | 1.477 | 0.075 | <0.0001 |
|  | C_D_H_M_ - C_D_C_M_ | 2.725 | 0.154 | <0.0001 |
|  | ΔD_d×m_ | -0.522 | 0.054 | <0.0001 |
| Direct breeding value of IBW | C_D_C_M_ | 5.4 | 2.1 | 0.009 |
| (g) | C_D_H_M_ | 30.2 | 3.1 | <0.0001 |
|  | H_D_C_M_ | 27.1 | 3.0 | <0.0001 |
|  | H_D_H_M_ | 34.2 | 2.1 | <0.0001 |
| Direct selection response of IBW | Group comparison | $\Delta D$ |  |  |
|  | H_D_C_M_ - C_D_C_M_ | 43.4 | 7.2 | <0.0001 |
|  | C_D_H_M_ - C_D_C_M_ | 99.2 | 15.0 | <0.0001 |
|  | ΔD_d×m_ | -17.7 | 5.2 | 0.02 |

Least squares mean (LSM) of direct breeding values for the various selection group and their comparisons used to estimate the selection responses. H and C represent high and control groups and the subscripts D and M denote direct and maternal genetic effects; $\Delta D$ direct selection response; ΔD_d×m_ are deviations from the expected response based on single effect selection as derived in equation (3); SVB, survival at birth, SVNP, survival during the nursing period, IBW, individual birth weight; SE standard error.

**Additional file 5 Table S5 Direct selection responses in survival and birth weight at the piglet level after adjustment of these traits for litter size**

| **Effect and trait** | **Selection group** | **LSM** | **SE** | **P-value** |
| --- | --- | --- | --- | --- |
| Direct breeding value of SVNP | C_D_C_M_ | 87.930 | 0.048 | <0.0001 |
| (%) | C_D_H_M_ | 88.817 | 0.071 | <0.0001 |
|  | H_D_C_M_ | 88.173 | 0.067 | <0.0001 |
|  | H_D_H_M_ | 87.819 | 0.048 | <0.0001 |
| Direct selection response of SVNP | Group comparison | $\Delta D$ |  |  |
|  | H_D_C_M_ - C_D_C_M_ | 0.485 | 0.165 | 0.010 |
|  | C_D_H_M_ - C_D_C_M_ | 3.546 | 0.341 | <0.0001 |
|  | ΔD_d×m_ | -1.240 | 0.119 | <0.0001 |
| Direct breeding value of SVB | C_D_C_M_ | 95.954 | 0.019 | <0.0001 |
| (%) | C_D_H_M_ | 96.319 | 0.029 | <0.0001 |
|  | H_D_C_M_ | 96.535 | 0.028 | <0.0001 |
|  | H_D_H_M_ | 96.465 | 0.020 | <0.0001 |
| Direct selection response of SVB | Group comparison | $\Delta D$ |  |  |
|  | H_D_C_M_ - C_D_C_M_ | 1.162 | 0.067 | <0.0001 |
|  | C_D_H_M_ - C_D_C_M_ | 1.461 | 0.139 | <0.0001 |
|  | ΔD_d×m_ | -0.435 | 0.049 | <0.0001 |
| Direct breeding value of IBW | C_D_C_M_ | 29.3 | 2.0 | <0.0001 |
| (g) | C_D_H_M_ | 31.4 | 3.0 | <0.0001 |
|  | H_D_C_M_ | 40.8 | 2.9 | <0.0001 |
|  | H_D_H_M_ | 22.5 | 2.0 | <0.0001 |
| Direct selection response of IBW | Group comparison | $\Delta D$ |  |  |
|  | H_D_C_M_ - C_D_C_M_ | 22.9 | 7.0 | <0.0001 |
|  | C_D_H_M_ - C_D_C_M_ | 8.2 | 14.5 | 1 |
|  | ΔD_d×m_ | -20.3 | 5.1 | 0.0001 |

Least squares mean (LSM) of direct breeding values for the various selection group and their comparisons used to estimate the selection responses. H and C represent high and control groups and the subscripts D and M denote direct and maternal genetic effects; $\Delta D$ direct selection response; ΔD_d×m_ are deviations from the expected response based on single effect selection as derived in equation (3); SVB, survival at birth, SVNP, survival during the nursing period, IBW, individual birth weight; SE standard error.
