# Supplementary material for: Evaluation of direct and maternal responses in reproduction traits based on different selection strategies for postnatal piglet survival in a selection experiment
Source: Genet Sel Evol. 2021 Mar 15;53:28. doi: 10.1186/s12711-021-00612-7 (PMC7958901; doi:10.1186/s12711-021-00612-7)
Supplement: Supplementary file 6 — Additional file 6: Figure S3. Generalised linear regression (fitting a probit link function) of survival at birth (SVB) on individual birthweight (IBW), including confidence interval at 95%. The figure shows the generalised linear regression (fitting a probit link function) of survival at birth (SVB) on individual birthweight (IBW), including confidence interval at 95%. Figure S4. Generalised linear regression (fitting a probit link function) of survival at birth (SVB) on individual birthweight (IBW), including confidence interval at 95%. The figure shows the generalised linear regression (fitting a probit link function) of survival during the nursing period (SVNP) on individual birthweight (IBW), including confidence interval at 95%. [file 12711_2021_612_MOESM6_ESM.docx]

**Additional file 6 Figure S3 Generalised linear regression (fitting a probit link function) of survival at birth (SVB) on individual birth weight (IBW), including confidence interval at 95%.**

**
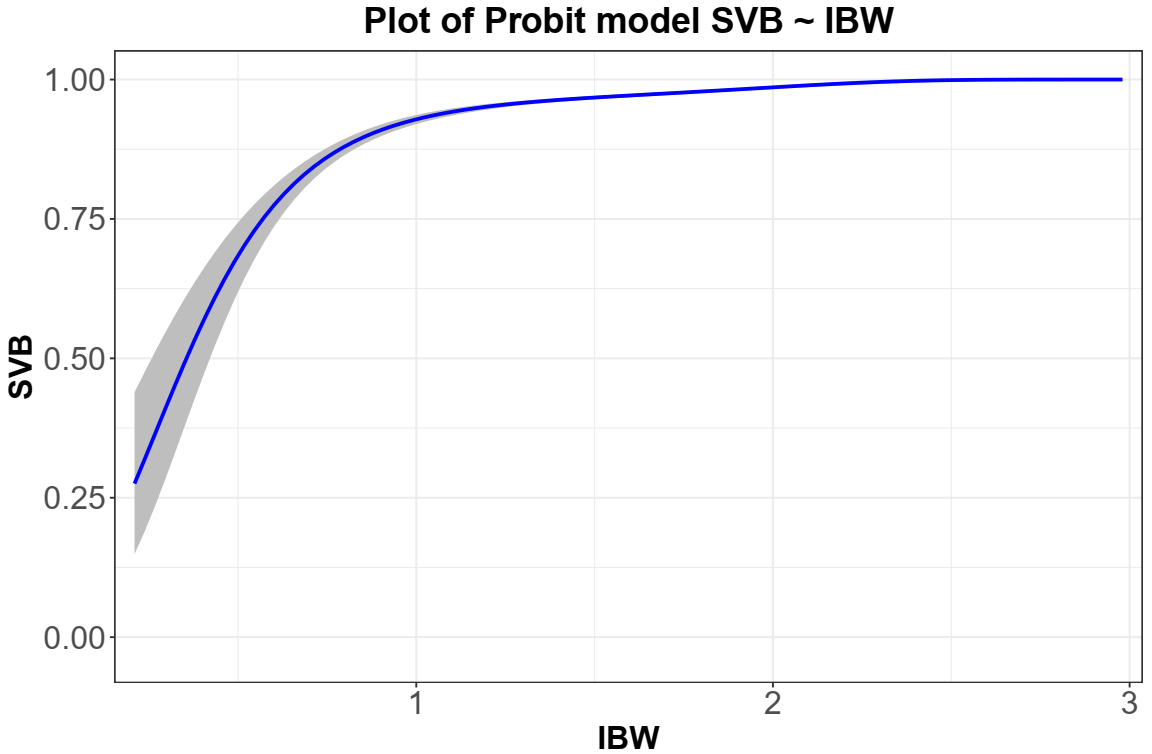
**

**Additional file 6 Figure S4 Generalised linear regression (fitting a probit link function) of survival during the nursing period (SVNP) on individual birth weight (IBW), including confidence interval at 95%.**

**
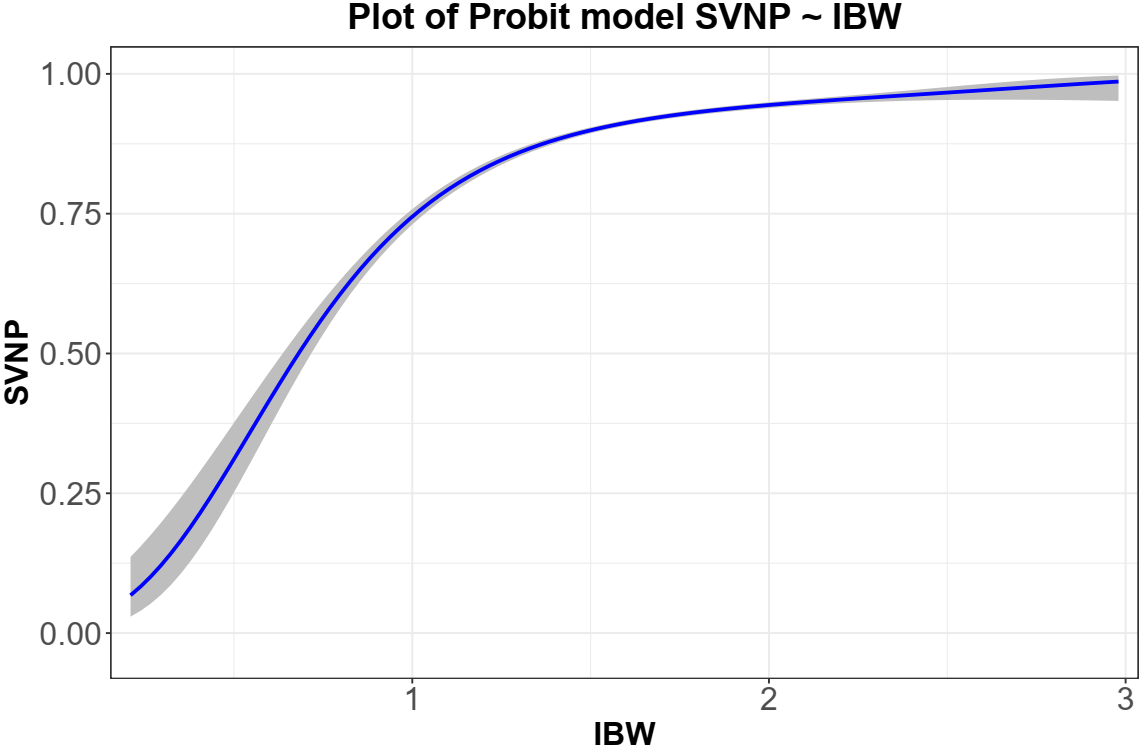
**
